# Mass Spectrometric and Bio-Computational Binding Strength Analysis of Multiply Charged RNAse S Gas-Phase Complexes Obtained by Electrospray Ionization from Varying In-Solution Equilibrium Conditions

**DOI:** 10.3390/ijms221910183

**Published:** 2021-09-22

**Authors:** Cornelia Koy, Kwabena F. M. Opuni, Bright D. Danquah, Andrei Neamtu, Michael O. Glocker

**Affiliations:** 1 Proteome Center Rostock, University Medicine Rostock and University of Rostock, Schillingallee 69, 18057 Rostock, Germany; cornelia.koy@med.uni-rostock.de (C.K.); kfopuni@ug.edu.gh (K.F.M.O.); danquahbright@yahoo.com (B.D.D.); 2 Department of Pharmaceutical Chemistry, School of Pharmacy, College of Health Science, University of Ghana, P.O. Box LG43, Legon, Ghana; 3 Department of Physiology, Grigore T. Popa University of Medicine and Pharmacy of Iasi, Str. Universitatii nr. 16, 700051 Iasi, Romania; neamtuandrei@gmail.com

**Keywords:** ESI-MS, desolvation process, ITEM-TWO, bio-computation, in-silico modeling, RNAse S, non-covalent complex, binding strength

## Abstract

We investigated the influence of a solvent’s composition on the stability of desorbed and multiply charged RNAse S ions by analyzing the non-covalent complex’s gas-phase dissociation processes. RNAse S was dissolved in electrospray ionization-compatible buffers with either increasing organic co-solvent content or different pHs. The direct transition of all the ions and the evaporation of the solvent from all the in-solution components of RNAse S under the respective in-solution conditions by electrospray ionization was followed by a collision-induced dissociation of the surviving non-covalent RNAse S complex ions. Both types of changes of solvent conditions yielded in mass spectrometrically observable differences of the in-solution complexation equilibria. Through quantitative analysis of the dissociation products, i.e., from normalized ion abundances of RNAse S, S-protein, and S-peptide, the apparent kinetic and apparent thermodynamic gas-phase complex properties were deduced. From the experimental data, it is concluded that the stability of RNAse S in the gas phase is independent of its in-solution equilibrium but is sensitive to the complexes’ gas-phase charge states. Bio-computational in-silico studies showed that after desolvation and ionization by electrospray, the remaining binding forces kept the S-peptide and S-protein together in the gas phase predominantly by polar interactions, which indirectly stabilized the in-bulk solution predominating non-polar intermolecular interactions. As polar interactions are sensitive to in-solution protonation, bio-computational results provide an explanation of quantitative experimental data with single amino acid residue resolution.

## 1. Introduction

Soft ionization methods, such as fast atom bombardment (FAB) [1], plasma desorption (PD) [2], laser desorption (LD) [3], matrix-assisted laser desorption/ionization (MALDI) [4], and electrospray ionization (ESI) [5,6] have been applied for mass spectrometric analyses of bio-macromolecules which were difficult or impossible to investigate when applying higher energy-transferring ionization techniques. From these soft ionization methods, ESI [7,8,9]—and to some extent MALDI [10,11,12]—have been suggested to potentially be capable of also studying non-covalent complexes. The correlation between protein compactness in solution and gas-phase charge structure of the ESI-generated protonated or de-protonated ions has been pointed out already in early reviews [13,14], suggesting that ESI was the ionization and desorption method of choice for investigating higher-order structures of bio-macromolecules by mass spectrometry.

One of the first ESI mass spectrometry investigations of non-covalent protein–peptide complexes made use of RNAse S [15,16], a protein quaternary structure that consists of the S-peptide and the S-protein. Since then, emphasis has been laid on choosing the appropriate solvent composition for stabilizing non-covalent complexes in solution as a prerequisite for qualitative analysis of the complexes’ ions’ charge structures [17,18]. Later, studies of ionized protein–protein complexes have been termed “native mass spectrometry” [12,19], branding a now broadly visible region of the structural biology research field [18,20] which has gained importance for determining key features of supramolecular assemblies of bio-macromolecules such as complex composition, the stoichiometry of complex constituents, identification of protein–protein binding interfaces (epitopes), and for analyzing gas-phase disassembly processes [21,22].

The direct transition of analytes from the condensed phase into the gas phase, i.e., desorption, has been recognized as the key process for successful mass spectrometric analysis of bio-macromolecules with desorption occurring shortly after or simultaneous to ionization [13,14]. Consequently, ESI processes that influenced the transition of intact non-covalent complexes into the gas phase as multiply charged supramolecular species have to be precisely controlled [23,24]. By keeping desorption and ionization under control, gas-phase complex monitoring methods might be regarded as equal to in-solution techniques for determining kinetic and thermodynamic properties of protein–protein complexes [22,25]. Upholding the vision to expand the applicability of ESI-MS to function as a prime tool for studying gas-phase stability, we have developed the ITEM approach [26], yielding in methods termed ITEM-ONE [26], ITEM-TWO [27], and ITEM-THREE [28], through which apparent kinetic and apparent thermodynamic properties of supramolecular complexes such as $k_{m0g}^{\#}$, $K_{D\;m0g}^{\#}$, and ${\Delta G}_{m0g}^{\#}$ can be determined [27,29].

In this report, we focus on investigating the influence of a solvent’s composition on the stability of desorbed and multiply charged RNAse S by collision-induced dissociation (CID) of the complexes’ ions. RNAse S was dissolved in different ESI-compatible aqueous buffers with either an increasing content of organic co-solvent or with low and neutral pH. The direct transition of all the in-solution equilibrium-constituting components, i.e., intact RNAse S and in-solution dissociated S-peptide and S-protein, was followed by (i) CID of the non-covalent RNAse S complex and (ii) by quantitative analysis of the dissociation products. From normalized ion abundances of all RNAse S-related molecular species were deduced the apparent kinetic and apparent thermodynamic gas-phase complex properties which in this report also include approximations of ${\Delta H}_{m0g}^{\#}$ and ${T\Delta S}_{m0g}^{\#}$. The experimentally determined complex stabilities are discussed in context with data obtained from computational bioinformatics, i.e., by in-silico investigations using the three-dimensional RNAse S structure which had been obtained by X-ray crystallography [30] or resulted from simulations when RNAse S had been dissolved in “bulk solution” or after release as a solvent-free multiply charged ion from “evaporating droplets”.

## 2. Results

### 2.1. RNAse S Gas Phase Dissociation Studies upon Electrospraying Protein Solutions with Different pHs

**Figure 1:**
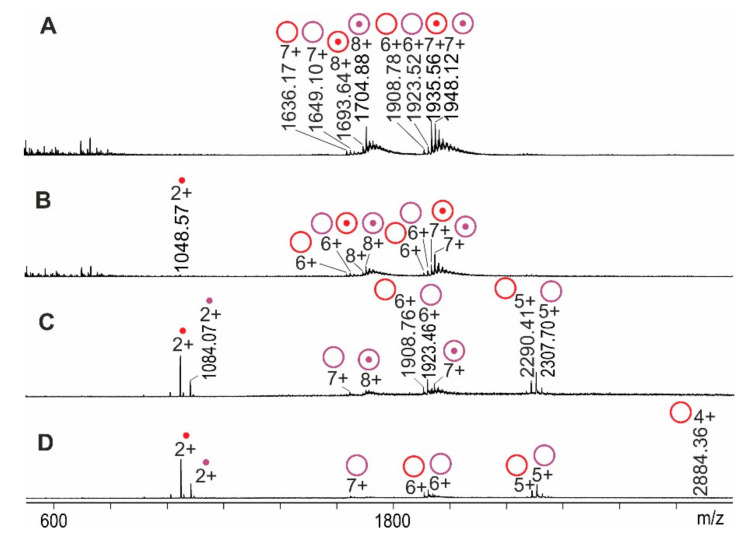
Nano-ESI mass spectra from RNAse S dissociation reactions. Collision cell voltage differences (∆*CV*): (**A**): 4 V. (**B**): 10 V. (**C**): 30 V. (**D**): 50 V. Charge states and *m*/*z* values for selected ion signals are given. RNAse S ions (reddish circles with dots), S-protein ions (reddish circles without dots), and released S-peptide ions (reddish dots) each represent the two major molecular species (red: low molecular weight species; purple: high molecular weight species). Solvent: 2% acetic acid/methanol (95:5, *v*/*v*) mixed with 200 mM ammonium acetate in 1:1 ratio, pH 4.5.

Like many non-covalent protein–peptide complexes, at a pH of 4.5, RNAse S was substantially dissociated into the S-protein and the S-peptide because of acidic solvent conditions. ESI mass spectra showed ion series for all molecular species from the in-solution equilibrium, i.e., RNAse S, S-protein, and S-peptide (Figure 1A). The average charge state of RNAse S was 7.3+ when dissolved at a pH of 4.5 (cf. Table 1).

The maximum charge of RNAse S was 8+ and that of the S-protein was 7+, whereas the S-peptide was doubly protonated at a pH of 4.5. The transmission of low-mass ions into later compartments of the mass spectrometer could be suppressed by adjusting quadrupole settings. Upon subjecting the mixture to CID, S-peptide ions dissociated from RNAse S ions which had traversed intact into the collision cell of the applied Q-ToF instrument. The step-by-step increase of the collision cell voltage difference, Δ*CV*, increased the intensities of product ions at the expense of educt ions´ intensities (Figure 1B–D and Supplementary Tables S1 and S2).

**Figure 2:**
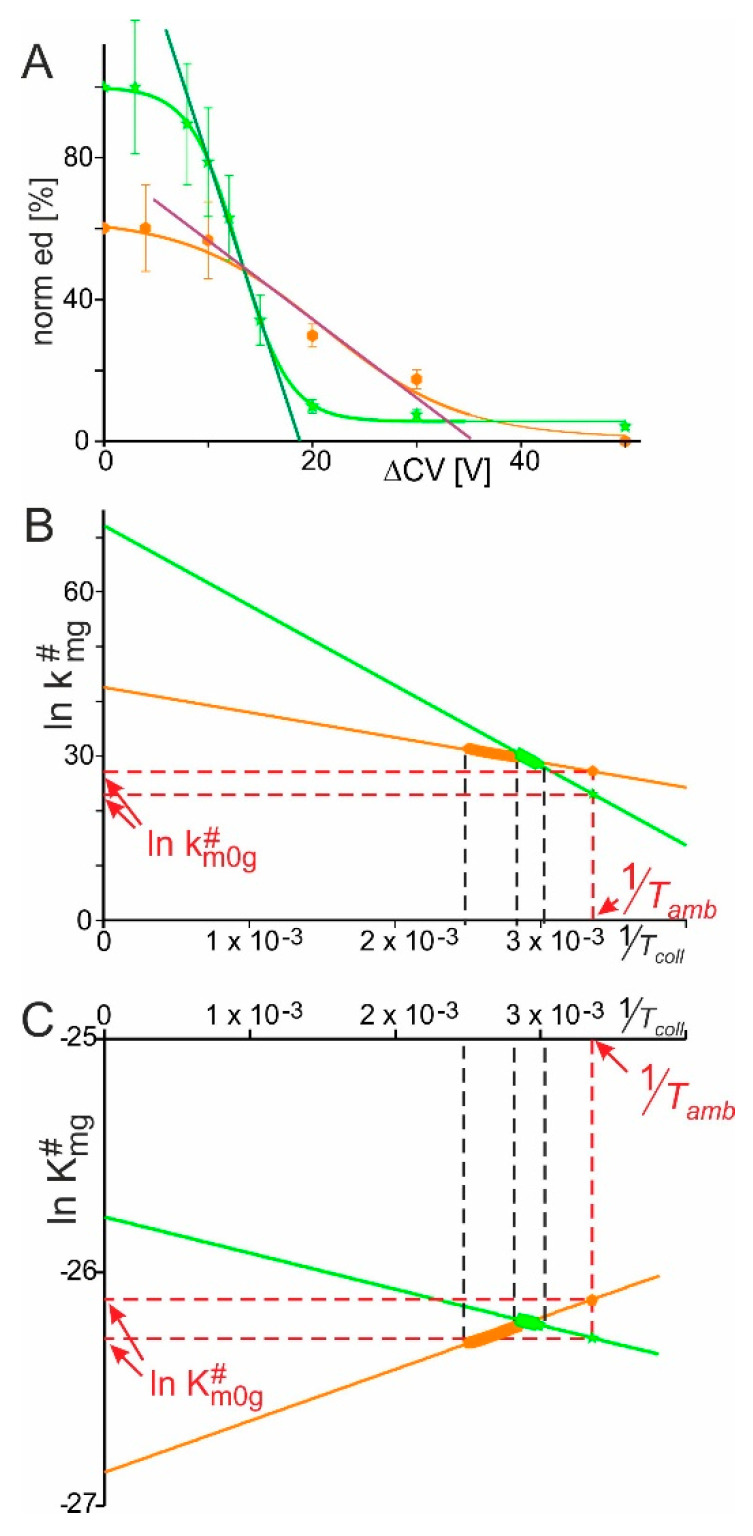
(**A**): Courses of normalized ion intensities of RNAse S ions ($norm\;\left(ed\right)$ ) as functions of collision cell voltage differences (ΔCV). Orange lines and data points: solution pH 4.5; green lines and data points: solution pH 7. Each data point is the mean of up to three independent measurements. Vertical bars give standard deviations. Curves were fitted using Boltzmann functions. Curve parameters used for calculations are listed in Table 1. Tangent lines are shown. (**B**): Arrhenius plots for the courses of RNAse S dissociation in the gas phase upon spraying at pH 4.5 or pH 7. Values for $\ln\;k_{m0g}^{\#}$ are taken from the points of the lines at $\frac{1}{T_{amb}}$. Line color code as in A. (**C**): Plots for the courses of RNAse S dissociation reactions in the gas phase over temperature. Line color code as in A. Each data point (thickened parts of the lines) is obtained from Equation (4) and the values for ${\ln\;K}_{D\;m0g}^{\#}$ are taken from the points of the lines at $\frac{1}{T_{amb}}$. The thickened parts of the lines in B and C depict the data points from the experimentally accessible ranges (black dashed lines). Calculated kinetic and thermodynamic values are listed in Table 2.

Ion intensities were determined by Gaussian fits over all the charge states of a given molecular species, and the heights of fitted curves’ apices, which represented molecular ion amounts, were averaged and normalized at each Δ*CV* setting. Plotting normalized educt ion intensities, i.e., RNAse S ion intensities, as a function of Δ*CV* provided sigmoidal-shaped curves with Boltzmann characteristics. The values from RNAse S dissolved in acidic solution yielded a normalized educt starting intensity rate of approximately 60% and intensity rates decreased steadily to nearly 0% (Figure 2A; for curve parameters see Table 1).

A distinctly different course of diminishing educt intensity rates with increasing Δ*CV* was observed when RNAse S was sprayed from the aqueous buffer at a pH of 7 (Supplementary Figure S1). The averaged and normalized educt intensity rates now started at ca. 100% (Table 1 and Supplementary Table S2). The 7+ charge states were the dominating ion signals whereas the 8+ charged ion signals barely existed when RNAse S was dissolved at a pH of 7. The average charge state of RNAse S was 6.9+ (cf. Table 1). Normalized educt intensity rates fell sharply to about 5% at approx. 13 V of Δ*CV* (Figure 2A, Table 1, and Supplementary Tables S3 and S4). The necessary values for generating Arrhenius plots were extracted from the Boltzmann curves, i.e., from the tangent lines along the steep parts of the curves (for details, see Section 5 and literature references therein). As in the Arrhenius plots $\ln\;k_{mg}^{\#}$ was linearly dependent on $\frac{1}{T_{\mathrm{coll}}}$ (Figure 2B), all apparent kinetic and thermodynamic physical quantities could be derived by applying the respective equations, i.e., the Arrhenius equation, the van’t Hoff equation, and the Eyring–Polanyi equation. As expected, from the Gibbs-Helmholtz equation courses of $\ln\;K_{mg}^{\#}$ were linearly dependent on $\frac{1}{T_{\mathrm{coll}}}$ as well (Figure 2C). Most strikingly, ${\Delta H}_{m0g}^{\#}$ of RNAse S, i.e., the free enthalpy of the RNAse S complex dissociation in the gas phase enabled a clear distinction. When RNAse S had been dissolved in aqueous buffer at a pH of 4.5, ${\Delta H}_{m0g}^{\#}$ was −1.83 kJ/mol, whereas ${\Delta H}_{m0g}^{\#}$ was +1.28 kJ/mol when RNAse S had been dissolved in an aqueous buffer at a pH of 7. A positive ΔH meant that heat was taken from the environment during the dissociation reaction (endothermic reaction), whereas a negative ΔH meant that heat was emitted during the dissociation reaction or into the environment (exothermic reaction).

### 2.2. RNAse S Gas Phase Dissociation Studies upon Electrospraying Protein Solutions with Different Content of Organic Co-Solvent

**Figure 3:**
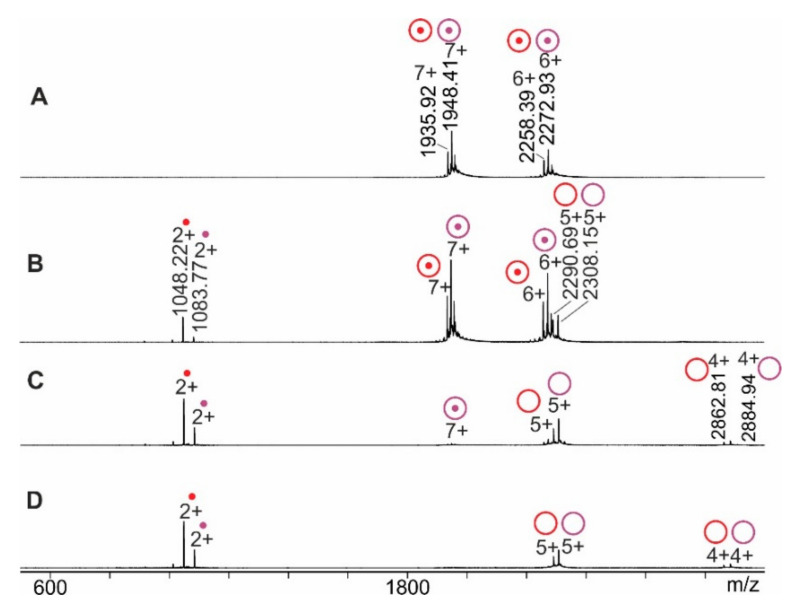
Nano-ESI mass spectra of RNAse S dissociation reactions. Collision cell voltage differences (∆*CV*): (**A**): 3 V. (**B**): 11 V. (**C**): 30 V. (**D**): 50 V. Charge states and *m*/*z* values for selected ion signals are given. RNAse S ions (reddish circles with dots), S-protein ions (reddish circles without dots), and released S-peptide ions (reddish dots) each represent the two major molecular species (red: low molecular weight species; purple: high molecular weight species). Solvent: 200 mM ammonium acetate/methanol (90:10, *v*/*v*), pH 7.

**Figure 4:**
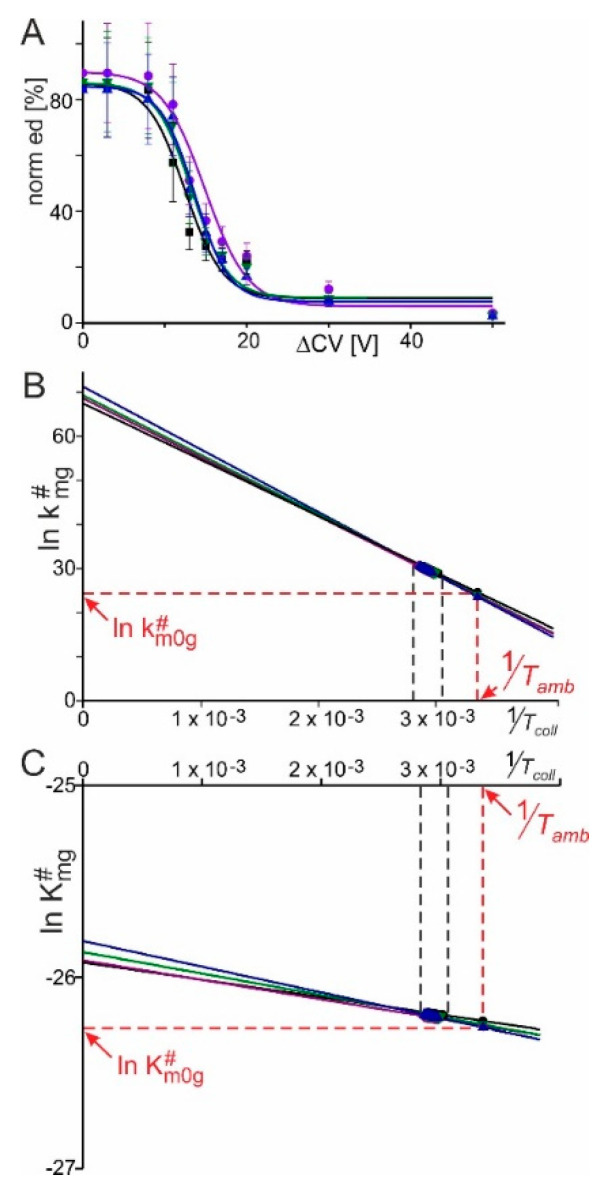
**(A**): Courses of normalized ion intensities of RNAse S ions ($norm\;\left(ed\right)$ ) as functions of collision cell voltage differences (ΔCV). Black lines and data points: 10% methanol; violet lines and data points: 20% methanol; dark green lines and data points: 30% methanol; dark blue lines and data points: 40% methanol. Each data point is the mean of up to three independent measurements. Vertical bars give standard deviations. Curves were fitted using Boltzmann functions. Curve parameters used for calculations are listed in Table 1. (**B**): Arrhenius plots for the courses of RNAse S dissociation in the gas phase upon spraying solutions with increasing methanol content. Values for $\ln\;k_{m0g}^{\#}$ are taken from the points of the lines at $\frac{1}{T_{amb}}$. Line color code as in A. (**C**): Plots for the courses of RNAse S dissociation reactions in the gas phase over temperature. Line color code as in A. Each data point (thickened parts of the lines) is obtained from Equation (4) and the values for ${\ln\;K}_{D\;m0g}^{\#}$ are taken from the points of the lines at $\frac{1}{T_{amb}}$. The thickened parts of the lines in B and C depict the data points from the experimentally accessible ranges (black dashed lines). Calculated kinetic and thermodynamic values are listed in Table 2.

As with changing pH, the in-solution equilibrium between intact RNAse S and its constituents, the S-protein and the S-peptide, was influenced by changing solvent conditions via increasing the content of organic co-solvent to the aqueous buffer. Mass spectra of RNAse S which was dissolved in 200 mM ammonium acetate/10% methanol showed 6+ and 7+ ion signals of intact RNAse S (Supplementary Figure S2), whereas with increasing methanol content additional ion signals appeared which belonged to the S-protein (6+) and the S-peptide (2+). The maximum amount of methanol was 40% in the described experiments in which the methanol content was raised by 10% in each step. In all cases, the average charge states of intact RNAse S were approximately 6.4+. Surviving multiply charged RNAse S ions from the mixtures of all protein constituents which existed in a solution in each of the respective methanol-containing solvents were subjected to CID which generated product ions by gas-phase dissociation (Figure 3 and Supplementary Figures S3–S5). Amounts of product ions, i.e., S-protein ions and S-peptide ions, increased steadily with increasing collision cell voltage difference, Δ*CV*, and plotting of normalized educt intensities as functions of Δ*CV* again provided sigmoidal-shaped Boltzmann curves in each case (Figure 4A and Table 1). Averaged and normalized educt intensity rates started at ca. 90% and 85%, respectively (Table 1). The values which were needed to generate Arrhenius plots (Figure 4B) were extracted from the Boltzmann curves as before (for details see Supplementary Tables S5–S12). The Gibbs–Helmholtz equation afforded more or less overlapping lines (Figure 4C) when extrapolating the experimentally accessible data over broader temperature regimes.

Apparent kinetic and approximated thermodynamic values for RNAse S dissociation after spraying from aqueous solutions with a neutral pH but with increasing methanol contents showed in principle the nearly same gas phase-dissociation behavior of RNAse S ions, independent from methanol content (Table 2). ${\Delta H}_{m0g}^{\#}$ was about +0.9 (±0.2) kJ/mol in all cases, leading to the conclusion that in all four investigated cases, gas phase-stability of RNAse S was independent of its respective *in-solution* equilibrium.

### 2.3. Bio-Computational Studies of RNAse S Conformations Forming during Electrospray Desolvation or in Bulk Solution

The ESI-MS desolvation process was modeled by first surrounding RNAse S in a sufficiently large virtual volume filled with water molecules, followed by the slow and gradual removal of water molecules during subsequent molecular dynamics simulations. During the simulation, the protonation state was switched from “bulk mode” (5+) to “droplet mode” (7+) before simulating the last 200 evaporation steps, i.e., when there were still 200 water molecules left on the proteins’ surface, almost forming a mono-molecular layer of solvent surrounding RNAse S. To take into account the higher gas-phase basicity of R vs. H vs. K residues, we first selected the 100 lowest energy states, then sorted them with respect to the number of protonated R residues, and finally chose the first ten richest R-protonated patterns to subject them to ten independent desolvation simulations. This procedure was termed the ‘Multiple-run-Multiple-States-Arg’ approach and was chosen since it better mimicked the experimental conditions where energetically quasi-degenerated protonation patterns may coexist for one and the same charge state but on different RNAse S conformers.

**Figure 5:**
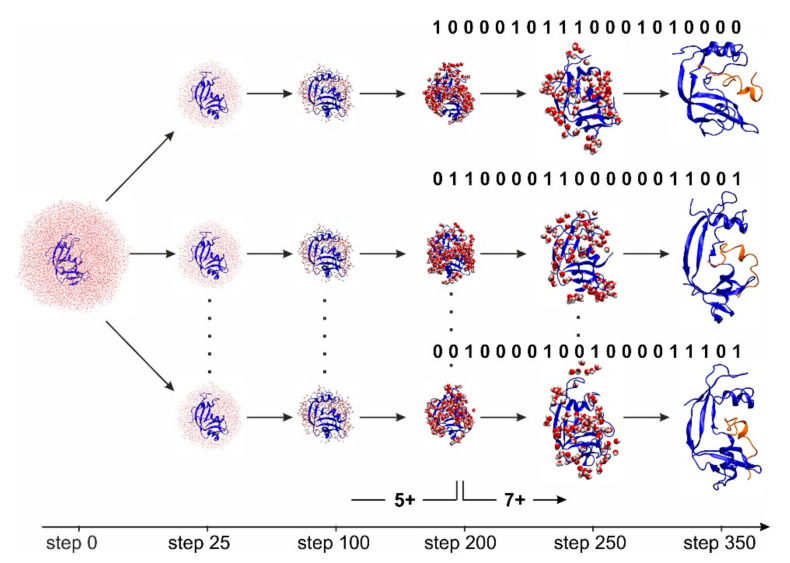
Snapshots of multiple-run-multiple-states-Arg (MMSA) simulations of the in-silico desolvation process of RNAse S. For each simulation with a total of 350 steps, each, a different proton location but an equal number of protons was set at step 200 of the simulation process to generate RNAse S ions with 7+ charge state patterns. 7+ charge state patterns were distributed over 20 protonatable sites as is indicated by the binary codes (1: occupied; 0: unoccupied). Solvent water molecules were removed after each step of the simulation by random selection (for details see text). The final RNAse S structures, three arbitrarily chosen structures are shown at the right, display subtle differences of amino acid residue positions, mostly affecting the S-peptide (red) which is kept bound to the S-protein (blue).

A major source of uncertainty in modeling ESI-MS evaporation and ionization events is the placement of the protons among all the acidic and basic sites of a protein. Since usually there are more basic side chains available in a protein amino acid sequence than the total charge of the protein requests, the number of protonation combinations that are compatible with a certain charge state can be tremendous. In the case of RNAse S, there are 20 protonatable basic sites (*n*) while the total charge of the complex is 7+ (*k*). This gives a total of $n!/\left(\left(n-k\right)!k!\right)=77,520$ possible charge patterns. For each protonatable residue, a value of ‘0’ (i.e., ‘not protonated’, charge 0) or ‘1’ (i.e., protonated, charge +1) was randomly generated in such a way that the total charge on the complex equaled the charge state with maximal intensity derived from the MS experiment, which at a pH of 7 is 7+. The obtained 20-digit binary string of ‘0’ and ‘1’ represents one individual protonation pattern. Analysis of bio-computational data was based on force-field simulations of RNAse S conformations which theoretically evolved in an evaporating droplet (Figure 5). Ten RNAse S conformations with minimal polar solvation energy and richest R-protonated states were picked from the set of 3 × 10^4^ models. Each of them was subjected to 0.05 µs simulation periods. Atom–atom distances between atoms from the S-peptide to those from the S-protein were determined for each model structure using the atom coordinates from the calculated RNAse S conformers (Supplementary Table S13). S-peptide amino acid residues 16–20/21 were not considered here as their positions were not resolved in the X-ray structure (1J80.pdb).

The numbers of contacts for each amino acid residue from the S-peptide were determined by setting a radius of 4 Å as the largest distance margin and yielded in “position-to-sum” ratios (see Section 5 for explanations). This ratio allows comparing numbers independent from residue size and independent from absolute numbers of contact possibilities for each residue. For all the ten models, the count of realized contact positions was 473. Most of the atom pair contacts between each amino acid residue from the S-peptide and each amino acid residue from the S-protein were listed just once i.e., they appeared in only one of the ten models.

The maximum number of models in which one and the same atom pair contact had been realized was three, providing a sum of 540 contacts over all the ten model structures. S-peptide amino acid residues with contact pairs which were realized in more than one RNAse S conformation were K7, F8, E9, R10, Q11, H12, M13, D14, and S15 (Supplementary Table S14). For these amino acid residues, the “position-to-sum ratios” were >1. By contrast, for the S-peptide amino acid residues K1, E2, T3, A4, A5, and A6, the “position-to-sum ratios” were 1.

Simulations of RNAse S conformations in “bulk solution” were performed for comparison and showed that when starting with the X-ray structure data (from 1KF5.pdb), RNAse S adopted slightly different conformations after 100 ns and 200 ns of simulation time, respectively (Supplementary Table S14). The S-peptide residues K1, E2, T3, A6, and K7 had no in-solution contacts to the S-protein and therefore their “position-to-sum ratios” were set to 0. S-peptide amino acid residues A4, A5, E9, R10, Q11, and S15 provided “position-to-sum ratios” of 1 and for S-peptide amino acid residues F8, H12, M13, and D14 “position-to-sum ratios” were >1. While S-peptide amino acid residues K1, E2, T3, A6, and K7 do not add directly to the strength of S-peptide—S-protein bonds in “bulk solution”, S-peptide amino acid residues A4, A5, E9, R10, Q11, and S15 do to some extent. At the same time, these amino acid residues experience some freedom of movement according to the simulated structure models. Of note, S-peptide amino acid residues F8, H12, M13, and D14 remain in “bulk solution” in close distance to their partner residues on the S-protein in all three structure models within the simulated time period.

### 2.4. Bio-Computational Studies on the Dependence of Complex Binding Strength on S-Peptide Amino Acid Residue Position

To further estimate which of the S-peptide amino acid residues may play key roles in binding to the S-protein, relative binding energy changes were determined upon site-specific exchanges of amino acid residues from the S-peptide. The wild-type amino acid residues in RNAse S were assigned as reference (Δ*G_bind_* = 0). Amino acid residues, which are different from the wild-type amino acid residue at a given position on the S-peptide sequence with higher binding energies, i.e., positive ΔΔ*G_bind_* values, were considered as having a destabilizing effect on the non-covalent interaction.

**Figure 6:**
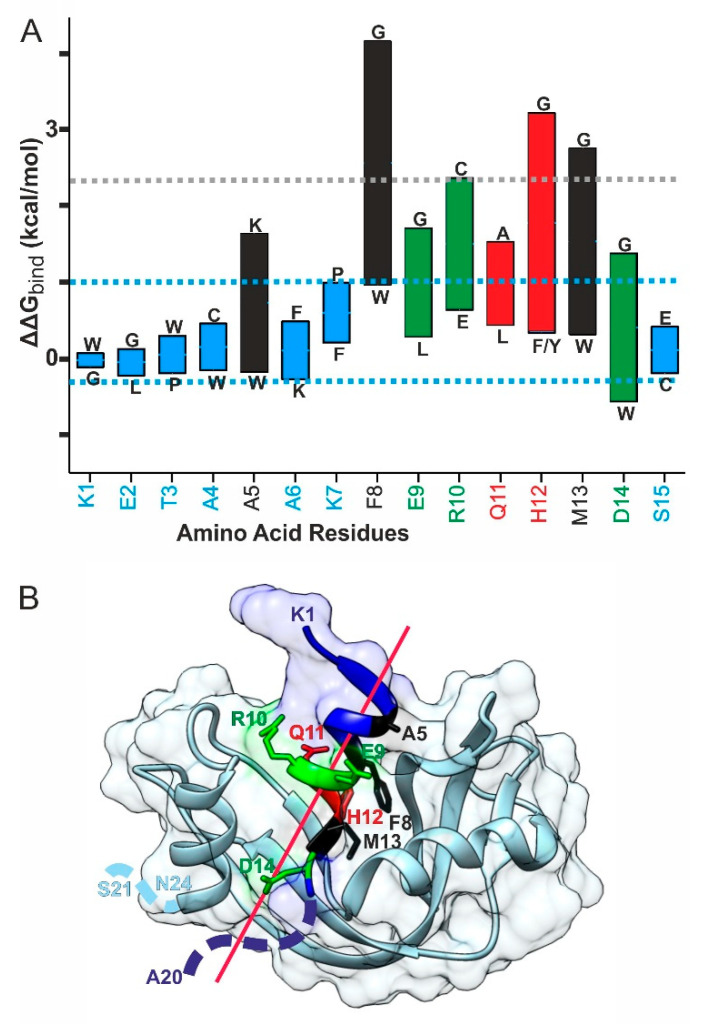
(**A**): Relative binding energy changes between S-peptide and S-protein upon site-specific exchange of amino acid residues of the S-peptide. Wild-type amino acid residues of the S-peptide are shown at the bottom. Black: non-polar residues; red: polar residues; green: ionic residues; blue: residues with no substantial influence on complex stability. Vertical bars show the ΔΔ*G_bind_* energy ranges after point mutations. Mutated amino acid residues with the highest and the lowest ΔΔ*G_bind_* values are shown above and below their respective bars. The dashed blue lines indicate borders within changes of ΔΔ*G_bind_* values do not cause significant stability changes. The dashed gray line indicates the border at twice the height of the range which spans from the bottom to the upper dashed blue line. (**B**): Three-dimensional structure representation of RNAse S. Atom position data are from 1J80.pdb. The atoms’ surfaces of the amino acid residues of the S-peptide (Connolly surfaces using van der Waals radii) are colored in blue whereas the S-protein surface is indicated in light blue. The backbones of the amino acid sequences of the S-peptide (blue) and the S-protein (light blue) are shown as ribbons (cartoon view). The side-chain atoms of the amino acid residues from the S-peptide which are involved in contacts with the S-protein are depicted as stick models. The red line marks the central axis of the S-peptide helix. Dashed blue lines indicate arbitrary backbone locations of amino acid residues for which no positions were stored in the PDB file. Amino acid residue color code as in (**A**).

By contrast, amino acid residues with negative ΔΔ*G_bind_* values were considered as providing a stabilizing effect (Figure 6A). The dashed blue lines indicate borders within which changes of ΔΔ*G_bind_* values did not cause significant stability changes, and the dashed gray line marks the border above the upper dashed blue line with the same distance as spanned between both blue dashed lines.

The binding energy variation analysis provided three types of S-peptide residues. No significant binding energy change was assigned to S-peptide residues K1, E2, T3, A4, A6, K7, and S15 as their energy changes, i.e., the respective ΔΔ*G_bind_* values, were within the dashed blue lines (Figure 6A). S-peptide amino acid residues with substantial energy changes upon exchanges with other amino acids were A5, E9, R10, Q11, and D14 because their ΔΔ*G_bind_* values crossed the upper blue dashed line but maxima were located below the dashed gray line (Figure 6A). At last, S-peptide amino acid residues with strong binding energy differences upon amino acid exchange, F8, H12, and M13, and energy changes, i.e., ΔΔ*G_bind_* values, reached beyond the dashed gray line (Figure 6A). In particular, the latter amino acid residues were considered crucial for binding in bulk solution. Of note, there is great overlap between amino acid residue groupings when distance variations were analyzed as compared to binding energy difference inspections. Like before, S-peptide amino acid residues 16–20/21 were not considered here as their positions were not resolved in the X-ray structure (Figure 6B).

To visualize the importance of individual amino acid residues of the S-peptide helix for binding to the S-protein, the amino acid residues were displayed in the three-dimensional structure representation of RNAse S (Figure 6B) which showed that the non-polar residues F8 and M13 together with the more polar residue H12 are located on the same side of the S-peptide helix which finds itself plugged into a pocket of the S-protein. These amino acid residues contribute critically to the non-covalent interaction that holds the S-peptide and the S-protein together. S-peptide amino acid residues on either its N-terminal end (residues 1–7 or its C-terminal end (residues 14–20/21), as well as residues on the outward-looking side of the S-peptide helix (residues 9–11) are part of the RNAse S surface with lesser or no substantial contribution to complex stability in “bulk solution”.

## 3. Discussion

Due to the rich data background on RNAse S, this commercially available non-covalent protein–peptide complex with clearly defined molecular composition is an ideal substance for developing methods by which supramolecular properties as well as influences of the solution environment can be elucidated in both the condensed and the gas phase. The desire to apply mass spectrometry for determining binding strengths of non-covalent forces, i.e., affinities between biomolecules was expressed already from early on [6] taking solution chemistry and biophysical concepts as models [24]. Our quantitative data on RNAse S dissociation in the gas phase substantiate the interpretation that interfering with polar interactions between S-peptide and S-protein by in-solution pH changes is resembled by a shift in mean charge states of the protein complex in the gas phase. This means that the pH-dependent protonation difference in solution is maintained in the gas phase and, thereby, affects the respective non-covalent complex stability. ITEM analysis showed that when RNAse S was dissolved at pH 4.5 the complex dissociated faster in the gas phase, i.e., upon adding lesser external energy as compared to RNAse S which had been dissolved at pH 7, which is consistent with molecular recognition studies in the gas phase [31].

Matching with this experimental observation, our in-silico simulations indicated that remaining forces that held the S-peptide complexed to the S-protein in the gas phase, i.e., after evaporation of solvent were consistent with previous estimations where subtle structural changes of protein surface amino acid residues (of protein complexes) in the gas phase were described to take place while simultaneously the protein ions maintained their over-all solution-like structures [24,32,33]. One limitation when choosing the force field for gas-phase simulations of protein structures is parametrization, as published parameter sets are available only for the neutral forms of amino acids. To our knowledge, only the OPLS-AA/L (2001) has the full consistent set of parameters for all the neutral forms of both, acidic and basic amino acid residues, including R residues, the neutral N-, and the neutral C-terminus. Therefore, OPLS-AA/L is considered best suitable by many simulation studies and also well applicable for gas-phase simulations of protein structures. For in-silico modeling of water molecules, the TIP4P-Ew set [34] was used as this parameter set is suitable for Ewald summation techniques when evaluating long-range electrostatic interactions. Also, at a certain stage during the droplet evaporation process the molecular topology, i.e., the protonation states of acidic/basic residues, had to be switched from the “bulk model”, i.e., the fully solvated protein, to the “droplet model”, i.e., that for the protein in the gas phase. Choosing the hydration level at which this charge transfer occurred was arbitrary to a large extent. There is evidence that even a few water molecules can stabilize zwitter-ionic forms and that the proton transfer that yields to the gas phase protonation state occurs at a relatively late stage of the evaporation process [35]. During our simulation procedure, we “slowed down” the loss of water after the 200th iteration to a loss of just one water molecule per simulation stage. This was done since we were more interested in sampling the protein structures from the final part of the evaporation process, i.e., when the local hydration conditions were far from that of the bulk phase and closer to that of the gas phase.

From our molecular dynamics simulations, an increase in the number of S-peptide to S-protein contacts was found for “outward” facing amino acid residues with no contacts to the S-protein in “bulk solution”, which is the case for K1, E2, A3, A6, and K7 (cf. Figure 6B). The respective conformational changes of these amino acid residues during evaporation of solvent are induced by gradual loss of solvation. As a consequence, the overall S-peptide to S-protein binding is strengthened by the respective conformational changes upon loss of solvation. It needs to be mentioned, that there is a loss of entropy associated by this process which opposes binding strength gain. However, from our data, it cannot be evaluated how important the molecular entropy loss is for the overall complex stability as on the other hand water molecules freed from solvation add to gain of entropy of the entire system. An increase in the number of molecule contacts is also possible for outward-facing amino acid residues on the S-peptide which simultaneously undergo some interactions to the S-protein already in “bulk solution”, which is exemplified with residues E9, R10, Q11, and S15. Again, a loss of solvation induced the respective conformational changes which added to the overall strengthening of the binding of the S-peptide to the S-protein; again, driven by a loss of solvation. No or only marginal change of the number of molecule contacts is seen for S-peptide amino acid residues A4 and A5 which were roughly facing towards the S-protein and may have been involved in binding of the S-peptide to the S-protein. But since even in “bulk solution” these residues did not provide strong binding forces, their contribution to the binding of the S-peptide onto the S-protein was also small in the gas phase. At last, a decrease in the number of molecule contacts was found for S-peptide amino acid residues which were facing right towards the S-protein and which were involved in strong binding of the S-peptide to the S-protein in “bulk solution”. The here to be mentioned amino acid residues are F8, H12, M13, and D14. With the exception of D14, these amino acid residues were involved mostly in hydrophobic interactions with S-protein residues. The respective contact changes of these residues were induced indirectly by loss of solvation. Ultimately, hydrophobic interactions lose their importance for intra- and intermolecular binding in a vacuum, i.e., when the solvent sphere had been lost. It should be mentioned that in the above-outlined considerations it is assumed that contact likelihood is equal for all listed atom-atom contacts.

Most interestingly, when in solution pH was changed, amino acid residues E9, R10, Q11, and S15 were expected to be affected the most because protonation interferes with the polar interactions in which these residues were involved. As a consequence, the overall S-peptide to S-protein binding was weakened by increasing the number of protons in the solution. Since protons keep moving around on the molecule surface during solvent evaporation, their impact on the binding strength of the S-peptide onto the S-protein is kept in the gas phase, which stands in agreement with both, the “salt bridge rearrangement” model [36] and the “unfolded chain ejection” model [37]. Of note, the situation was different when the in-solution equilibrium of RNAse S was altered by adding organic co-solvent. Then, the charge state of the RNAse S complex was not altered and, therefore, all surviving RNAse S was kept together in the gas phase in the same manner. This leads to the conclusion that in all four investigated cases, the gas phase-stability of RNase S was independent of its respective in-solution equilibrium.

## 4. Conclusions and Outlook

In sum, the combination of in-silico analyses of simulated structures in both, “bulk solution” and in an “evaporating droplet” with experimental data obtained by ESI-MS helped to gain a deeper understanding of the non-covalent gas-phase forces which play important roles to keep non-covalent protein complexes together. As both, the polar and non-polar non-covalent binding forces survived the transition of the intact RNAse S complex from solution into the gas phase they could be nullified by “ITEM” experiments in the gas phase, which ultimately led to dissociation of the S-peptide from intact RNAse S by applying different CID energies. The requested CID forces thereby provided measurable physical quantities for the RNAse S complex stability in the gas phase and at the same time reflected important in-solution properties of the non-covalent protein complex. As the roles of individual amino acid residues could be interrogated for their contributions they added to hold the S-peptide onto the S-protein of RNAse S, the “ITEM”-method should be considered rather powerful for investigating the importance of amino acid substitutions on binding strength differences, as long as such “mutated” complexes were sprayed from identical solutions under constant ESI-MS conditions as well.

With the advent of therapeutic antibodies for patient treatment, there is a huge demand for antibody characterization, both structurally and functionally. As the most specific property of an antibody is arguably its ability to bind its antigen through paratope—epitope recognition, the two prime objectives of characterizing a therapeutic antibody are (i) to identify the epitopes and (ii) to estimate the strength of antibody—antigen binding. Information provided by results of epitope mapping experiments is extremely valuable in the process of antibody humanization as well as in vaccine design. Of similar importance, rapid diagnostic tests are first-line assays for diagnosing infectious diseases such as Covid-19. To minimize false positive and false negative test results in population screening assays, high-quality reagents and well-characterized antigens and antibodies are needed. Furthermore, “key amino acid residues” concerning antigen—antibody binding within epitope peptides ought to be studied to determine which mutations in the identified epitope would be tolerated without harming stable immune complex formation. This information is of importance when mutations on the antigen have to be taken into consideration, e.g., when fearing immune evasion. Such information can be retrieved by determining the binding strength similarities or differences between the “wild-type” epitope and a “mutated” epitope.

## 5. Material and Methods

### 5.1. Preparation of NanoESI-MS Compatible RNAse S Solutions

A stock solution of RNAse S and bovine pancreas (1 µg/µL) was prepared by dissolving the lyophilized protein powder (Sigma-Aldrich, Steinheim, Germany; Lot No. 52H7034) in 200 mM ammonium acetate buffer, pH 7.0 [29]. An aliquot of 100 µL was transferred onto a centrifugal filter (Microcon, 3 kDa cut-off, Millipore Corporation, Bedford, MA, USA) and a further volume of 200 µL of 200 mM ammonium acetate buffer was added. This solution was centrifuged at 13,000 rpm for 30 min at 23 °C using the MIKRO 22R centrifuge (Hettich GmbH & Co., Tuttlingen, Germany). The eluate was discarded and 200 µL of 200 mM ammonium acetate buffer was added to the retentate. Centrifugation followed as described above. This procedure, i.e., re-filling of retentate, centrifugation, and discarding of eluate, was repeated three times. Then, the filter unit was inverted and placed into a new tube. Centrifugation at 4500 rpm for 5 min afforded a volume of approximately 70 µL which was collected, and the protein concentration of this solution was determined using the Qubit™ 2.0 Fluorometer (Carlsbad, California, USA) assay [38]. Typical RNAse S concentrations were between 0.4 µg/µL and 0.8 µg/µL. For nano electrospray mass spectrometry, the re-buffered RNAse S solution was diluted to a final concentration of 0.2 µg/µL. A solvent mixture that consisted of 5% up to 40% (*v*/*v*) methanol which was dissolved in 200 mM ammonium acetate buffer was used as diluent. To obtain an RNAse S containing acidic solution with final protein concentration of 0.2 µg/µL and pH 4.5, 10 µL of a solution with 2% acetic acid/methanol (95:5, *v*/*v*) was added to 10 µL of RNAse S (0.4 µg/µL) which was dissolved in 200 mM ammonium acetate buffer.

### 5.2. Nanospray Needle Preparation

NanoESI needles for off-line measurements were prepared in-house as previously described [26]. Briefly, borosilicate glass tubes with an inner diameter of 0.5 mm and an outer diameter of 1.0 mm were used to produce the needles using a P-1000 Flaming/Brown Micropipette Puller System (Sutter Instrument, Novato, CA, USA). Capillary needles were gold-coated under argon atmosphere utilizing the SCD005 Sputter Coater (BAL-TEC AG, Balzers, Liechtenstein) by setting the following parameters: current 20 mA, sputter time duration 150 s, 5 cm working distance of the needles to the gold foil target, and argon gas pressure 0.5 mbar.

### 5.3. Off-Line NanoESI-MS Instrument Settings and Data Acquisition Conditions

For each measurement, ca. 3 µL of the respective freshly prepared RNAse S solution were loaded into gold-coated nanoESI capillary needles using microloader pipette tips (Eppendorf, Hamburg, Germany). Mass spectra were acquired on a Q-ToF 2 instrument (Waters MS-Technologies, Manchester, UK). ITEM-TWO measurements [27] were performed with the following instrument settings: capillary needle voltage, 1.1 kV; sample cone voltage, 20 V; extractor cone voltage, 3 V; RF lens voltage 1.2 V, source temperature, 40 °C; collision voltage, 3 V; pusher time, 88 µs. The quadrupole and ToF analyzer pressures were typically between ca. 2.0 × 10^−5^ mbar and 2.50 × 10^−7^ mbar, respectively. All mass spectra were acquired in positive-ion mode within a mass-to-charge window of *m*/*z* 500–3000. The *m*/*z* axis was calibrated using a solution of orthophosphoric acid (1%) dissolved in 50% TFE. At the starting point of the ITEM-TWO experiment, the quadrupole analyzer was set to block transmission of ions below *m*/*z* 1600 by choosing the settings: M1 = 1750 with dwell time of 5% and ramp time of 5%; M2 = 1750 with dwell time of 5% and ramp time of 85%; M3 = 1750. Next, the collision gas was switched on and set to 1.2 bar. Three RNAse S measurement series were acquired for each solvent in which RNAse S had been dissolved in (10% to 40% methanol/200 mM ammonium acetate, pH 7, or 5% methanol at pH 4.5 and pH 7, respectively) and spectra were recorded at the respective collision cell voltage differences (Δ*CV*), except for RNAse S in 20% methanol/200 mM ammonium acetate where only two datasets were recorded. For every measurement series, Δ*CV* steps were 3 or 4, 8, 10 or 11, 13, 15, 17, 20, 30, and 50 V. At each Δ*CV* setting, mass spectra were recorded for 3 min each. The combined scans for each Δ*CV* setting were taken to generate an average mass spectrum using the MassLynx software version 4.0 (Waters MS-Technologies, Manchester, UK). Each average mass spectrum from every Δ*CV* setting was smoothed using the Savitzky–Golay method in 6 cycles with a window of 12 for the experiments at 10% to 40% methanol/200 mM ammonium acetate buffer. Spectra that were acquired at pH 4.5 and pH 7, respectively, were smoothed with the Savitzky–Golay method in 10 cycles with a window of 10. Ion signals at the respective *m*/*z* values were assigned with respective charge states to the appropriate RNAse S complexes, S-proteins, and S-peptides. The mass spectrometry data have been deposited to the ProteomeXchange Consortium via the PRIDE [39] partner repository with the dataset identifier PXD027723.

### 5.4. Mass Spectral Data Analysis and Calculation of Kinetic and Thermodynamic Values of RNAse S

The smoothed mass spectra were used to determine heights of all multiply charged ion signals of RNAse S complexes (educts), S-proteins, and S-peptides (products) at all applied Δ*CV*, which were separately determined and subjected to Gaussian fitting. At each Δ*CV* setting the apices of the fitted Gaussian curves, i.e., maximum intensities and respective average *m*/*z* values for each molecular ion series, were determined using the Origin software (Origin Lab Corporation, Northampton, MA, USA; version 2018b). Subsequently, normalized relative amounts of educts and of products were calculated based on the nanoESI mass spectral data at each Δ*CV* setting. Then, plots of normalized intensities of educts (RNAse S) vs. Δ*CV* values were fitted to Boltzmann curves with regression coefficients of R^2^ ≥ 0.98, as described earlier [29]. The equations which were applied for calculations of the physical quantities $k_{m0g}^{\#}$, $K_{D\;m0g}^{\#}$, and ${\Delta G}_{m0g}^{\#}$ can be found in our earlier publications as well [27,29].

For calculating ${\Delta H}_{m0g}^{\#}$ and ${T\Delta S}_{m0g}^{\#}$ the Gibbs–Helmholtz equation (Equation (1)) is adapted and applied as follows:

(1) $${\Delta G}_{\mathrm{mg}}^{\#}={\;\Delta H}_{\mathrm{mg}}^{\#}-{\;T}_{\mathrm{coll}}{\;\Delta S}_{\mathrm{mg}}^{\#}$$

where:

$∆G_{\;mg}^{\#}$ change of Gibbs energy of activation to transform educt ions into product ions
$∆H_{\;mg}^{\#}$ heat change between educt ions and product ions
$T∆S_{\;mg}^{\#}$ change of disorder of educt ions and product ions
  (#: apparent; m: mean charge state; g: gas phase)

Also, by applying the van’t Hoff equation (Equation (2)) in the adapted version, $\Delta G_{mg}^{\#}$ can be described as:

(2) $${\Delta G}_{\mathrm{mg}}^{\#}=-{R\;T}_{\mathrm{coll}}\;\ln K_{D\;\mathrm{mg}}^{\#}\;$$

where:

$∆G_{\;mg}^{\#}$ change of Gibbs energy of activation
R gas constant, 8.314 J/mol·K
$T_{\mathrm{coll}}$ educt and/or product temperature during collision in the collision cell
$K_{D\;mg}^{\#}$ gas phase thermodynamic equilibrium dissociation constant

Inserting Equation (2) into Equation (1) results in Equation (3):

(3) $$-{\;R\;T}_{\mathrm{coll}}\;\ln K_{D\;\mathrm{mg}}^{\#}={\;\Delta H}_{\mathrm{mg}}^{\#}-{\;T}_{\mathrm{coll}}{\;\Delta S}_{\mathrm{mg}}^{\#}$$

Equation (3) is converted to Equation (4):

(4) $$\ln K_{D\;\mathrm{mg}}^{\#}=-\frac{{\Delta H}_{\mathrm{mg}}^{\#}}{R}\;*\frac{1}{T_{\mathrm{coll}}}+\frac{{\Delta S}_{\mathrm{mg}}^{\#}}{R}$$

Plotting ${\ln K}_{D\;\mathrm{mg}}^{\#}$ as a function of $\frac{1}{T_{\mathrm{coll}}}$ leads to a line with slope *–* $\frac{{\Delta H}_{\mathrm{mg}}^{\#}}{R}$ and *y*-axis intercept at $\frac{{\Delta S}_{\mathrm{mg}}^{\#}}{R}$. Because the slope of the line remains the same all along the line, the slope at point $\frac{1}{T_{amb}}$ is defined as $\frac{{\Delta H}_{m0g}^{\#}}{R}$, which at point $\frac{1}{T_{amb}}$ leads to Equation (5):

(5) $$\frac{{\Delta H}_{\mathrm{mg}}^{\#}}{R}=\frac{{\Delta H}_{m0g}^{\#}}{R}$$

where:

$∆H_{\;m0g}^{\#}$ heat change between educt ions and product ions
  (#: apparent; m: mean charge state; 0: no external energy contribution; g: gas phase)
$T_{amb}$ absolute ambient temperature, 298 K.

Because *R* is the gas constant, 8.314 J/mol·K, from $\frac{{\Delta H}_{m0g}^{\#}}{R}$ is calculated ${\;\Delta H}_{m0g}^{\#}$.

Again, because *R* is the gas constant, 8.314 J/mol·K, and $T_{amb}$ is the absolute ambient temperature, 298 K, from $\frac{\Delta S_{m0g}^{\#}}{R}$ is calculated $T_{amb}\;\Delta S_{m0g}^{\#}$. 

### 5.5. In-Silico Simulation Methodology for Estimating RNAse S Conformational Changes upon Desolvation

The atom coordinates of RNAse A, stored as 1KF5.pdb data set [40], were downloaded from the Protein Data Bank [41] and served as starting structure for in-silico generation of an RNAse S working molecular model. The RNAse S model was generated by deleting the peptide bond which in RNAse A connected residues A20 with S21 and by adding the atoms of one water molecule at the cleavage sites, thereby separating the S-peptide from the S-protein. Then, the RNAse S structure model was immersed in spherical droplets containing 10,000 water molecules each. These virtual assemblies were subjected to in-silico evaporation process simulations at 20 °C to generate RNAse S conformers which evolved in “evaporating droplets” within 50 ns each. The RNAse S charge state mode in the gas phase was taken from experiment as being 7+ at pH 7. To cope with the high dimensionality problem of placing 7 positive charges randomly onto 20 protonatable basic sites on RNAse S, Monte Carlo algorithms were applied to find the protonation states with minimized Coulomb electrostatic energies [42,43]. An automated algorithm was developed for exploring the protonation pattern, resulting in a randomly generated ensemble of 3 × 10^4^ of such charge distribution patterns. For each charge pattern, an appropriately protonated molecular RNAse S model structure was constructed and subjected to an initial geometry optimization, followed by 100 ps molecular dynamics runs at 1 K to ‘thermalize’ the protein. A second geometry optimization followed and in the end, the models were evaluated by Poisson–Boltzmann polar solvation energy calculations [44]. The evaporation process was modelled using 10 structure models, each representing one of the richest Arg protonated states. Each of these was subjected to 350 sequential dehydration steps. In each step a predefined number of water molecules was removed, and the resulting system was subjected to molecular dynamics simulations for equilibration to the emerging conditions. The water molecules to be removed were randomly selected from the entire number of water molecules present in the system at a given time. Each molecular dynamics run started with new random velocities taken from a Maxwell distribution at the simulation temperature. The number of water molecules which were removed in each step was not equal: larger numbers of water molecules were removed per step at the beginning of the evaporation process compared to the later stages. For the last 200 dehydration steps, only one molecule of water was removed at a time. The simulation time of each step was gradually increased, yielding in a total time of 50 ns for each of the 10 independent simulations. The protonation state was switched from “bulk mode” to “droplet mode” before simulating the last 200 evaporation steps when there were still 200 water molecules left on the protein surface, almost forming a mono-molecular layer of solvent surrounding RNAse S.

As reference, conformational rearrangements of fully solvated RNAse S were simulated for 200 ns at 20 °C while RNAse S was placed in a virtual periodic boundaries cubic box of solvent consisting of 10,000 water molecules, mimicking “bulk solvation” conditions. Both total charge and protonation pattern of RNAse S in solution were predicted using pKa calculations [45]. A total charge of 5+ was predicted for the bulk mode solvated RNAse S at pH 7. All simulations were performed using the GROMACS 2019 simulation suite [46]. The OPLS-AA/L force field with 2001 refitted torsional parameters was applied to both structure simulation projects [47]. All structure models were stored as PDB files for further analysis.

### 5.6. Visualization of RNAse S Structure and Calculation of Inter-Residue Contacts

The coordinates of all non-hydrogen atoms of the X-ray structure of the RNAse S complex (PDB file ID: 1J80, [30] were downloaded from the RCSB Protein Data Bank (available online https://www.rcsb.org (accessed on 7 February 2021)) and analyzed for atom–atom contacts of S-peptide amino acid residues with S-protein amino acid residues. Likewise, the atom coordinates from the in-silico molecular dynamics simulations were subjected to inspection for atom–atom distances of the amino acid residues of the S-peptide which made contact with atoms from amino acid residues of the S-protein. The RNAse S protein structures were visualized using the UCSF Chimera (available online http://www.cgl.ucsf.edu/chimera/ (accessed on 19 March 2021)) molecular visualization software [48]. The atoms of the amino acid residues of the S-peptide which made contact with atoms from the S-protein within a distance of ≤4 Å were determined using the UCSF Chimera software [49].

Calculations of “position-to-sum” ratios were performed in three steps: (i) for a given residue all listed contacts were added (sum of contacts), (ii) the number of their contacts in all the structure models which were investigated from the respective data set were counted (position contacts), and (iii) from the two sums we calculated ratios called “position to sum ratios”. Obviously, this ratio makes the numbers to be compared independent from the size of the residue and the absolute number of contact possibilities which would be dependent on the number of atoms of a respective residue. It also makes the numbers to be compared independent on the number of simulations which were investigated.

### 5.7. Calculation of Binding Energy Changes of S-Peptide Amino Acid Residue Substitutions

The binding energy changes between the S-peptide and the S-protein of RNAse S upon site-specific exchanges of amino acid residues from the S-peptide were performed using the BeAtMuSiC web server (available online http://babylone.ulb.ac.be/beatmusic/query.php (accessed on 15 February 2021)) [49,50]. The PDB file of the RNAse S complex (PDB ID: 1J80, [30]) served as the input file. The S-peptide and the S-protein were selected as the two binding partners. The server automatically performed systematic mutations of interface residues of the atoms belonging to the S-peptide. Data were visualized using the Origin software (Origin Lab Corporation, Northampton, MA, USA; version 2018b) [49,51].

## Figures and Tables

**Table 1:** Course parameters of gas-phase dissociation of RNAse S determined from mass spectra.

pH/MeOH [-] ^(a)^/[vol. %]	Mean Charge ± Std. Dev. ^(a)^	Initial Educt Rate [%] ^(b)^	Final Educt Rate [%] ^(b)^	∆*CV*_50_ [V]	dx [V]	Slope [%/V]
4.5/5	7.3 ± 0.1	63.00	0.84	21.10	6.59	−2.36
7/5	6.9 ± 0.1	100.00	5.60	13.01	2.37	−9.94
7/10	6.4 ± 0.2	86.00	8.77	12.50	2.30	−8.39
7/20	6.1 ± 0.6	90.00	5.99	14.90	2.60	−8.08
7/30	6.4 ± 0.2	86.00	9.04	13.30	2.20	−8.75
7/40	6.5 ± 0.2	84.75	7.65	13.66	2.08	−9.28

^(a)^ dimensionless number; ^(b)^ normalized.

**Table 2:** Apparent kinetic and thermodynamic values for gas-phase dissociation of RNAse S.

pH/MeOH [-] ^(a,b)^/[vol. %]	$k_{m0g}^{\#}$ [1/s]	$K_{D\;m0g}^{\#}\;[-]{\;}^{\left(b\right)}$	$\Delta G_{m0g}^{\#}\;[\mathrm{kJ}/\mathrm{mol}]$	$\Delta H_{m0g}^{\#}\;[\mathrm{kJ}/\mathrm{mol}]$	$T\Delta S_{m0g}^{\#}\;[\mathrm{kJ}/\mathrm{mol}]$
4.5/5	6.90 × 10^11^	4.39 × 10^−12^	64.79	−1.83	−66.54
7/5	1.03 × 10^10^	3.71 × 10^−12^	65.21	+1.28	−63.83
7/10	5.00 × 10^10^	3.97 × 10^−12^	65.04	+0.76	−64.22
7/20	2.09 × 10^10^	3.83 × 10^−12^	65.13	+0.84	−64.20
7/30	2.80 × 10^10^	3.87 × 10^−12^	65.10	+0.94	−64.09
7/40	1.71 × 10^10^	3.79 × 10^−12^	65.15	+1.12	−63.95

^(a)^ neutral and resting complex. ^(b)^ dimensionless number. # apparent.

## Data Availability

The mass spectrometry data have been deposited to the ProteomeXchange Consortium via the PRIDE [39] partner repository with the dataset identifier PXD027723.

## Supplementary Materials

The following are available online at https://www.mdpi.com/article/10.3390/ijms221910183/s1.

## Abbreviations

FAB	fast atom bombardment
PD	plasma desorption
LD	laser desorption
MALDI	matrix-assisted laser desorption/ionization
ESI	electrospray ionization
ITEM	intact transition epitope mapping
ITEM-ONE	ITEM- One-step Non-covalent force Exploration
ITEM-TWO	ITEM- Thermodynamic Weak-force Order
ITEM-THREE	ITEM- Targeted High-Energy Rupture of Extracted Epitopes
CID	collision induced dissociation
TFE	trifluoroethanol
TIC	total ion current
RNAse S	ribonuclease S
ASA	accessible surface area
PDB	protein database
PRIDE	proteomics identifications database
Q-ToF	quadrupole time-of-flight
Δ*CV*	collisional voltage difference
*m*/*z*	mass-to-charge ratio
