# Supplementary material for: Mass Spectrometric and Bio-Computational Binding Strength Analysis of Multiply Charged RNAse S Gas-Phase Complexes Obtained by Electrospray Ionization from Varying In-Solution Equilibrium Conditions
Source: Int J Mol Sci. 2021 Sep 22;22(19):10183. doi: 10.3390/ijms221910183 (PMC8508491; doi:10.3390/ijms221910183)
Supplement: Supplementary file 1 [file ijms-22-10183-s001.zip › ijms-1368946-supplementary.pdf]

Mass spectrometric and bio-computational binding strength analysis of multiply charged RNAse S gas phase complexes obtained by electrospray ionization from varying *in-solution* equilibrium conditions.

Cornelia Koy[1], Kwabena F. M. Opuni[1,2], Bright D. Danquah[1], Andrei Neamtu[3], Michael O. Glocker[1]

[1]Proteome Center Rostock, University Medicine Rostock and University of Rostock, Schillingallee 69, 18059 Rostock, Germany
[2]Department of Pharmaceutical Chemistry, School of Pharmacy, College of Health Science, University of Ghana, P. O. Box LG43 Legon, Ghana
[3]Department of Physiology, Gr. T. Popa University of Medicine and Pharmacy of Iasi, Str. Universitatii nr. 16, Iasi Jud., Romania

Supplement

Corresponding author

Prof. Dr. Michael O. Glocker
Proteome Center Rostock
University Rostock Medical Center and Natural Science Faculty
University of Rostock
Schillingallee 69
18057 Rostock
Germany

Phone: +49 - 381 - 494 4930
FAX: +49 - 381 - 494 4932
e-mail: michael.glocker@med.uni-rostock.de
URL: https://pzr.med.uni-rostock.de

Supplemental Figures

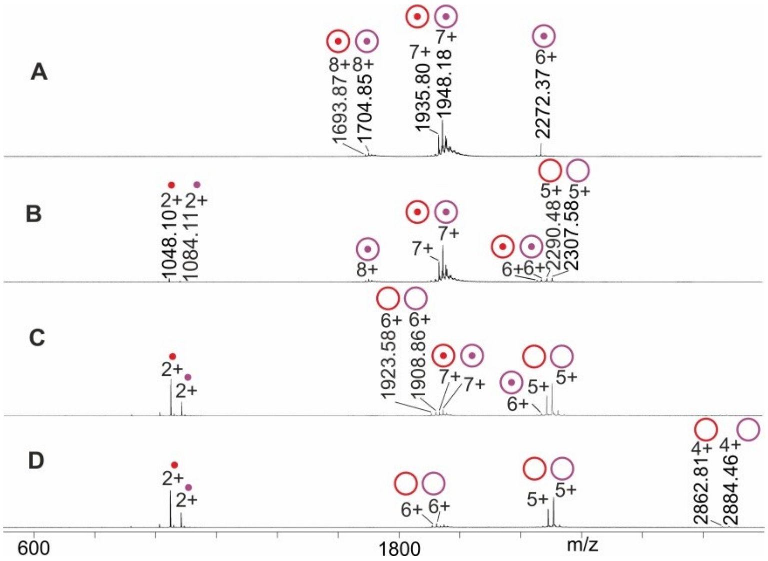

Supplemental Figure 1. Nano-ESI mass spectra from RNAse S dissociation reactions. Collision cell voltage differences (ΔCV): **A:** 3 V. **B:** 10 V. **C:** 30 V. **D:** 50 V. Charge states and *m/z* values for selected ion signals are given. RNAse S ions (reddish circles with dots), S-protein ions (reddish circles without dots) and released S-peptide ions (reddish dots) represent two major molecular species, each (red: low molecular weight species; purple: high molecular weight species). Solvent: 200 mM ammonium acetate / methanol (95:5, v/v), pH 7.

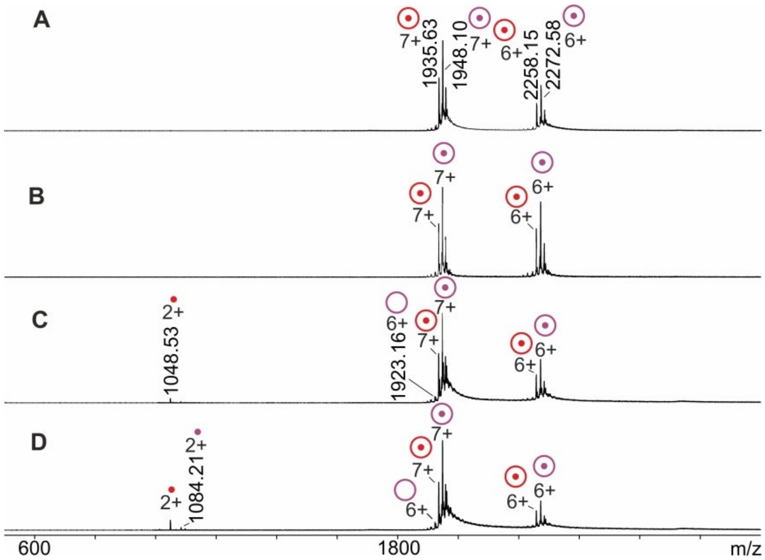

Supplemental Figure 2. Nano-ESI mass spectra from RNAse S prior to gas phase dissociation. RNAse S was dissolved in 200 mM ammonium acetate / methanol with increasing methanol concentrations. **A:** 10 %. **B:** 20 %. **C:** 30 %. **D:** 40 %. Charge states and *m/z* values for selected ion signals are given. RNAse S ions (reddish circles with dots), S-protein ions (reddish circles without dots) and released S-peptide ions (reddish dots) represent two major molecular species, each (red: low molecular weight species; purple: high molecular weight species).

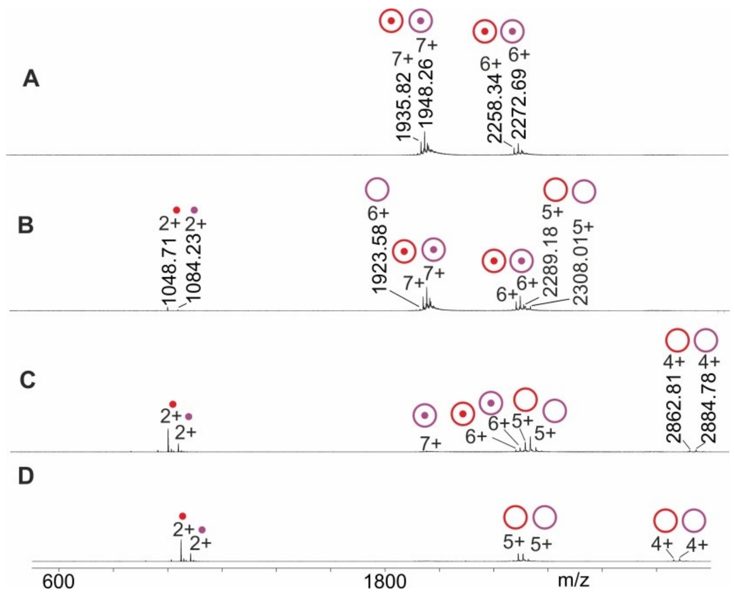

Supplemental Figure 3. Nano-ESI mass spectra of RNAse S dissociation reactions. Collision cell voltage differences (ΔCV): **A:** 3 V. **B:** 11 V. **C:** 30 V. **D:** 50 V. Charge states and *m/z* values for selected ion signals are given. RNAse S ions (reddish circles with dots), S-protein ions (reddish circles without dots) and released S-peptide ions (reddish dots) represent two major molecular species, each (red: low molecular weight species; purple: high molecular weight species). Solvent: 200 mM ammonium acetate / methanol (80:20, v/v), pH 7.

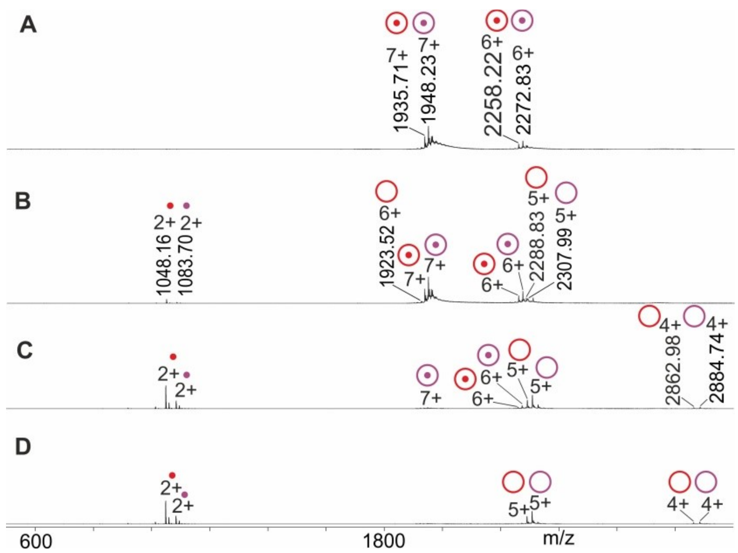

Supplemental Figure 4. Nano-ESI mass spectra of RNAse S dissociation reactions. Collision cell voltage differences (ΔCV): **A:** 3 V. **B:** 11 V. **C:** 30 V. **D:** 50 V. Charge states and *m/z* values for selected ion signals are given. RNAse S ions (reddish circles with dots), S-protein ions (reddish circles without dots) and released S-peptide ions (reddish dots) represent two major molecular species, each (red: low molecular weight species; purple: high molecular weight species). Solvent: 200 mM ammonium acetate / methanol (70:30, v/v), pH 7.

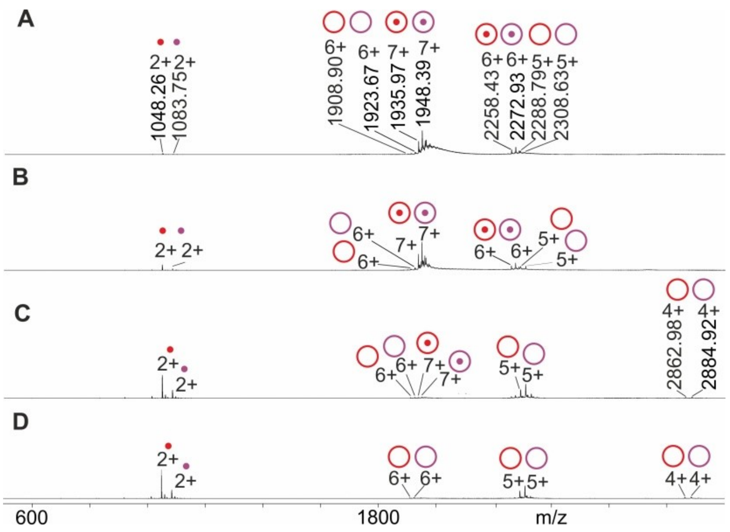

Supplemental Figure 5. Nano-ESI mass spectra of RNAse S dissociation reactions. Collision cell voltage differences (ΔCV): **A:** 3 V. **B:** 11 V. **C:** 30 V. **D:** 50 V. Charge states and *m/z* values for selected ion signals are given. RNAse S ions (reddish circles with dots), S-protein ions (reddish circles without dots) and released S-peptide ions (reddish dots) represent two major molecular species, each (red: low molecular weight species; purple: high molecular weight species). Solvent: 200 mM ammonium acetate / methanol (60:40, v/v), pH 7.

Supplemental Table 1: Ion intensities, charge states, and m/z values for RNAse S at various collision cell voltage difference settings in 5% methanol / 5% acetic acid in 200 mM ammonium acetate (pH 4.5).

1st determination

RNAse S Complex 1-19...21-124

ion	m/z	4V	10V	20V	30V	50V
5+	2727.08	0.00	0.00	0.00	0.00	0.00
6+	2272.78	0.00	1.25	0.00	0.00	0.00
7+	1948.17	8.14	11.50	3.44	4.47	0.00
8+	1704.79	5.03	6.07	0.00	2.46	0.00
9+	1515.49	0.00	0.00	0.00	0.00	0.00

RNAse S Complex 1-20...22-124

ion	m/z	4V	10V	20V	30V	50V
5+	2709.58	0.00	0.00	0.00	0.00	0.00
6+	2258.15	0.00	0.00	0.00	0.00	0.00
7+	1935.70	5.03	7.37	2.16	3.25	0.00
8+	1693.85	2.66	3.22	0.00	0.00	0.00
9+	1505.76	0.00	0.00	0.00	0.00	0.00

S Protein 21-124

ion	m/z	4V	10V	20V	30V	50V
3+	3845.75	0.00	0.00	0.00	0.00	0.00
4+	2884.67	0.00	0.00	0.00	0.00	0.00
5+	2307.79	0.00	1.90	4.26	9.37	15.10
6+	1923.36	1.55	2.79	2.56	6.17	9.05
7+	1648.75	1.60	1.85	0.00	1.79	2.56

S Protein 22-124

ion	m/z	4V	10V	20V	30V	50V
3+	3816.46	0.00	0.00	0.00	0.00	0.00
4+	2862.59	0.00	0.00	0.00	2.50	2.24
5+	2290.29	0.00	0.00	2.79	6.16	7.67
6+	1908.72	0.00	1.97	1.67	4.30	7.47
7+	1636.20	1.00	1.40	0.00	1.33	2.20

S Peptide 1-19

ion	m/z	4V	10V	20V	30V	50V
1+D	4189.30	0.00	0.00	0.00	0.00	0.00
1+	2095.15	0.00	0.00	0.00	0.00	0.00
2+	1048.07	1.00	1.50	6.39	19.70	41.70
3+	699.05	5.00	5.84	2.96	1.00	1.00
4+	524.53	0.00	0.00	0.00	0.00	0.00

S Peptide 1-20

ion	m/z	4V	10V	20V	30V	50V
1+D	4330.36	0.00	0.00	0.00	0.00	0.00
1+	2166.18	0.00	0.00	0.00	0.00	0.00
2+	1083.60	0.00	0.00	2.19	6.75	14.20
3+	722.72	1.92	1.73	1.22	0.50	0.50
4+	542.30	0.00	10.00	0.00	0.00	0.00

D=Dimer

Supplemental Table 1: continued

2nd determination

RNAse S Complex 1-19...21-124						
ion	m/z	4V	10V	20V	30V	50V
5+	2727.08	0.00	0.00	0.00	0.00	0.00
6+	2272.78	1.84	2.89	0.00	0.00	0.00
7+	1948.17	25.00	27.10	6.65	9.80	0.00
8+	1704.79	12.50	12.40	2.83	5.27	0.00
9+	1515.49	0.00	0.00	0.00	0.00	0.00

RNAse S Complex 1-20...22-124						
ion	m/z	4V	10V	20V	30V	50V
5+	2709.58	0.00	0.00	0.00	0.00	0.00
6+	2258.15	1.51	0.00	0.00	0.00	0.00
7+	1935.70	14.20	14.50	5.82	5.89	0.00
8+	1693.85	6.96	5.80	1.94	3.10	0.00
9+	1505.76	0.00	0.00	0.00	0.00	0.00

S Protein 21-124						
ion	m/z	4V	10V	20V	30V	50V
3+	3845.75	0.00	0.00	0.00	0.00	0.00
4+	2884.67	0.00	0.00	0.00	0.00	2.48
5+	2307.79	0.00	2.10	6.72	18.90	29.00
6+	1923.36	6.77	7.68	5.61	13.20	18.20
7+	1648.75	0.00	1.00	0.00	1.00	1.00

S Protein 22-124						
ion	m/z	4V	10V	20V	30V	50V
3+	3816.46	0.00	0.00	0.00	0.00	0.00
4+	2862.59	0.00	0.00	0.00	0.00	0.00
5+	2290.29	0.00	2.86	5.30	12.20	16.20
6+	1908.72	4.60	4.77	3.63	7.27	11.70
7+	1636.20	3.21	3.56	0.00	2.41	2.81

S Peptide 1-19						
ion	m/z	4V	10V	20V	30V	50V
1+D	4189.30	0.00	0.00	0.00	0.00	0.00
1+	2095.15	0.00	0.00	0.00	0.00	0.00
2+	1048.07	1.18	2.54	11.70	31.30	79.90
3+	699.05	11.30	11.30	4.69	1.00	2.60
4+	524.53	0.00	0.00	0.00	0.00	0.00

S Peptide 1-20						
ion	m/z	4V	10V	20V	30V	50V
1+D	4330.36	0.00	0.00	0.00	0.00	0.00
1+	2166.18	0.00	0.00	0.00	0.00	0.00
2+	1083.60	0.00	0.00	3.98	12.20	30.00
3+	722.72	5.00	5.26	0.00	0.00	0.00
4+	542.30	0.00	0.00	0.00	0.00	0.00

D=Dimer

Supplemental Table 1: continued

3rd determination

RNAse S Complex 1-19...21-124						
ion	m/z	4V	10V	20V	30V	50V
5+	2727.08	0.00	0.00	0.00	0.00	0.00
6+	2272.78	2.10	2.76	0.00	0.00	0.00
7+	1948.17	25.10	24.70	8.11	10.10	0.00
8+	1704.79	13.00	12.10	3.45	6.14	0.00
9+	1515.49	0.00	0.00	0.00	0.00	0.00

RNAse S Complex 1-20...22-124						
ion	m/z	4V	10V	20V	30V	50V
5+	2709.58	0.00	0.00	0.00	0.00	0.00
6+	2258.15	1.06	2.45	0.00	0.00	0.00
7+	1935.70	13.30	15.10	5.33	6.73	0.00
8+	1693.85	8.66	6.20	0.00	3.96	0.00
9+	1505.76	0.00	0.00	0.00	0.00	0.00

S Protein 21-124						
ion	m/z	4V	10V	20V	30V	50V
3+	3845.75	0.00	0.00	0.00	0.00	0.00
4+	2884.67	0.00	0.00	0.00	0.00	0.00
5+	2307.79	0.00	2.76	8.28	18.50	26.40
6+	1923.36	6.25	6.88	4.46	13.10	20.80
7+	1648.75	3.72	4.79	0.00	3.09	4.93

S Protein 22-124						
ion	m/z	4V	10V	20V	30V	50V
3+	3816.46	0.00	0.00	0.00	0.00	0.00
4+	2862.59	0.00	0.00	0.00	0.00	0.00
5+	2290.29	0.00	0.00	6.16	11.90	17.80
6+	1908.72	0.00	4.98	4.24	7.11	13.50
7+	1636.20	0.00	3.11	0.00	2.68	3.93

S Peptide 1-19						
ion	m/z	4V	10V	20V	30V	50V
1+D	4189.30	0.00	0.00	0.00	0.00	0.00
1+	2095.15	0.00	0.00	0.00	0.00	0.00
2+	1048.07	1.19	2.80	11.90	32.10	85.40
3+	699.05	12.40	11.50	3.77	1.00	2.40
4+	524.53	0.00	0.00	0.00	0.00	0.00

S Peptide 1-20						
ion	m/z	4V	10V	20V	30V	50V
1+D	4330.36	0.00	0.00	0.00	0.00	0.00
1+	2166.18	0.00	0.00	0.00	0.00	0.00
2+	1083.60	0.00	0.00	4.98	10.80	31.40
3+	722.72	5.15	4.70	1.48	0.00	1.00
4+	542.30	0.00	0.00	0.00	0.00	0.00

D=Dimer

Supplemental Table 2: Apex heights and mean charge states of educt and product ion signals upon gas phase dissociation of RNAse S dissolved in 5% MeOH / 5% AcOH in 200 mM ammonium acetate (pH 4.5).

1st determination [a)]

**ΔCV**	RNAse S				S-Protein				S-Peptide			
**[V]**	1-19..21-124		1-20..22-124		21-124		22-124		1-19		1-20	
	$m_{(1)}$+	$h_{(1)}$	$m_{(2)}$+	$h_{(2)}$	$p_{(1)}$+	$j_{(1)}$	$p_{(2)}$+	$j_{(2)}$	$n_{(1)}$+	$i_{(1)}$	$n_{(2)}$+	$i_{(2)}$
**4**	7.4	8.25	7.4	5.19	6.5	1.65	7.4	1.05	2.8	4.96	3.0	1.93
**10**	7.3	12.00	7.3	7.55	6.0	2.79	7.3	1.94	2.8	5.88	3.9	10.15
**20**	7.0	3.43	7.0	2.15	5.4	4.23	7.0	2.83	2.3	6.34	2.4	2.20
**30**	7.4	4.62	7.0	3.24	5.6	9.40	7.0	6.30	2.0	19.97	2.1	6.80
**50**	-	0.00	-	0.00	5.5	14.97	-	7.72	2.0	42.67	2.0	14.48

[a)] arbitrary units; cf. Figure 1

2nd determination [a)]

**ΔCV**	RNAse S				S-Protein				S-Peptide			
**[V]**	1-19..21-124		1-20..22-124		21-124		22-124		1-19		1-20	
	$m_{(1)}$+	$h_{(1)}$	$m_{(2)}$+	$h_{(2)}$	$p_{(1)}$+	$j_{(1)}$	$p_{(2)}$+	$j_{(2)}$	$n_{(1)}$+	$i_{(1)}$	$n_{(2)}$+	$i_{(2)}$
**4**	7.3	25.59	7.2	14.56	6.0	6.73	6.4	4.56	2.9	11.44	3.0	5.02
**10**	7.2	27.79	7.3	14.70	5.9	7.75	6.1	4.81	2.8	11.39	3.0	5.29
**20**	7.3	6.74	7.3	5.96	5.5	6.64	5.4	5.39	2.3	11.60	2.0	4.10
**30**	7.4	9.96	7.3	5.99	5.5	19.13	5.6	12.16	2.0	31.95	2.0	12.58
**50**	-	0.00	-	0.00	5.4	29.72	5.6	16.30	2.0	81.55	2.0	30.10

[a)] arbitrary units; cf. Figure 1

3rd determination [a)]

**ΔCV**	RNAse S				S-Protein				S-Peptide			
**[V]**	1-19..21-124		1-20..22-124		21-124		22-124		1-19		1-20	
	$m_{(1)}$+	$h_{(1)}$	$m_{(2)}$+	$h_{(2)}$	$p_{(1)}$+	$j_{(1)}$	$p_{(2)}$+	$j_{(2)}$	$n_{(1)}$+	$i_{(1)}$	$n_{(2)}$+	$i_{(2)}$
**4**	7.3	25.71	7.3	13.27	6.4	6.26	7.3	0.00	2.9	12.55	3.0	5.18
**10**	7.2	25.33	7.2	15.18	6.1	6.87	7.2	4.95	2.8	11.71	3.0	4.72
**20**	7.3	8.23	7.0	5.34	5.4	8.27	7.0	6.23	2.2	12.02	2.2	4.99
**30**	7.4	10.29	7.4	6.76	5.6	18.52	7.4	11.98	2.0	32.58	2.0	10.95
**50**	-	0.00	-	0.00	5.6	26.27	-	17.72	2.0	86.24	2.0	31.97

[a)] arbitrary units; cf. Figure 1

Supplemental Table 3: Ion intensities, charge states, and m/z values for RNAse S at various collision cell voltage difference settings in 5% methanol/200 mM ammonium acetate (pH 7).

1st determination

RNAse S Complex 1-19...21-124									
ion	m/z	3V	8V	10V	12V	15V	20V	30V	50V
5+	2727.08	0.00	0.00	0.00	0.00	0.00	0.00	0.00	0.00
6+	2272.78	4.20	6.43	7.82	8.34	9.72	8.11	8.16	6.48
7+	1948.17	54.50	81.30	83.40	83.50	58.90	18.60	21.70	15.30
8+	1704.79	4.09	6.21	6.42	5.58	2.89	1.00	1.79	1.31
9+	1515.49	0.00	0.00	0.00	0.00	0.00	0.00	0.00	0.00

RNAse S Complex 1-20...22-124									
ion	m/z	3V	8V	10V	12V	15V	20V	30V	50V
5+	2709.58	0.00	0.00	0.00	0.00	0.00	0.00	0.00	0.00
6+	2258.15	2.81	4.84	4.38	5.46	5.34	5.21	3.75	2.28
7+	1935.70	29.30	45.80	48.30	47.50	31.40	11.20	11.80	8.57
8+	1693.85	1.70	3.10	3.44	2.76	1.92	1.00	1.00	1.00
9+	1505.76	0.00	0.00	0.00	0.00	0.00	0.00	0.00	0.00

S Protein 21-124									
ion	m/z	3V	8V	10V	12V	15V	20V	30V	50V
3+	3845.75	0.00	0.00	0.00	0.00	0.00	0.00	0.00	0.00
4+	2884.67	0.00	0.00	0.00	0.00	0.00	0.00	2.78	4.24
5+	2307.79	0.00	4.69	11.50	22.20	44.80	69.00	107.00	127.00
6+	1923.36	0.00	6.63	7.69	9.92	12.90	9.24	14.10	18.20
7+	1648.75	0.00	1.00	1.00	0.50	1.00	0.40	1.80	2.34

S Protein 22-124									
ion	m/z	3V	8V	10V	12V	15V	20V	30V	50V
3+	3816.46	0.00	0.00	0.00	0.00	0.00	0.00	0.00	0.00
4+	2862.59	0.00	0.00	0.00	0.00	0.00	0.00	2.50	2.24
5+	2290.29	0.00	3.26	7.54	15.80	31.90	45.10	66.80	74.50
6+	1908.72	0.00	2.31	3.02	4.46	6.03	4.56	7.61	10.60
7+	1636.20	0.00	0.00	0.00	0.43	0.70	0.00	1.80	0.70

S Peptide 1-19									
ion	m/z	3V	8V	10V	12V	15V	20V	30V	50V
1+D	4189.30	0.00	0.00	0.00	0.00	0.00	0.00	0.00	0.00
1+	2095.15	0.00	0.00	0.00	0.00	0.00	0.00	0.00	0.00
2+	1048.07	0.00	4.94	12.30	25.60	64.10	107.00	181.00	195.00
3+	699.05	0.00	1.00	1.24	1.15	1.66	2.61	1.00	0.50
4+	524.53	0.00	0.00	0.00	0.00	0.00	0.00	0.00	0.00

S Peptide 1-20									
ion	m/z	3V	8V	10V	12V	15V	20V	30V	50V
1+D	4330.36	0.00	0.00	0.00	0.00	0.00	0.00	0.00	0.00
1+	2166.18	0.00	0.00	0.00	0.00	0.00	0.00	0.00	0.00
2+	1083.60	0.00	1.58	3.43	9.18	20.20	42.60	65.20	80.40
3+	722.72	0.00	0.42	0.40	0.40	0.40	1.22	0.00	0.00
4+	542.30	0.00	0.00	0.00	0.00	0.00	0.00	0.00	0.00

D=Dimer

Supplemental Table 3: continued

2nd determination

RNAse S Complex 1-19...21-124									
ion	m/z	3V	8V	10V	12V	15V	20V	30V	50V
5+	2727.08	0.00	0.00	0.00	0.00	0.00	0.00	0.00	0.00
6+	2272.78	4.33	7.12	7.12	8.84	9.99	8.38	8.17	5.42
7+	1948.17	67.60	94.80	100.00	90.20	60.40	23.10	20.80	11.40
8+	1704.79	6.78	8.23	8.90	6.52	3.08	1.00	1.15	0.50
9+	1515.49	0.00	0.00	0.00	0.00	0.00	0.00	0.00	0.00

RNAse S Complex 1-20...22-124									
ion	m/z	3V	8V	10V	12V	15V	20V	30V	50V
5+	2709.58	0.00	0.00	0.00	0.00	0.00	0.00	0.00	0.00
6+	2258.15	2.79	4.42	5.49	4.91	5.73	5.41	3.49	1.88
7+	1935.70	39.90	56.60	55.30	53.40	34.00	12.30	13.10	6.73
8+	1693.85	4.04	4.07	4.32	2.72	1.63	1.00	1.00	0.00
9+	1505.76	0.00	0.00	0.00	0.00	0.00	0.00	0.00	0.00

S Protein 21-124									
ion	m/z	3V	8V	10V	12V	15V	20V	30V	50V
3+	3845.75	0.00	0.00	0.00	0.00	0.00	0.00	0.00	0.00
4+	2884.67	0.00	0.00	0.00	0.00	0.00	0.00	2.75	4.54
5+	2307.79	0.00	5.58	11.20	27.90	60.50	83.30	111.00	120.00
6+	1923.36	0.00	7.13	8.66	10.80	12.40	8.45	13.10	16.00
7+	1648.75	0.00	0.00	1.00	1.03	1.00	0.00	1.00	1.00

S Protein 22-124									
ion	m/z	3V	8V	10V	12V	15V	20V	30V	50V
3+	3816.46	0.00	0.00	0.00	0.00	0.00	0.00	0.00	0.00
4+	2862.59	0.00	0.00	0.00	0.00	0.00	5.41	2.32	3.01
5+	2290.29	0.00	3.46	10.00	18.10	37.50	50.00	70.40	72.70
6+	1908.72	0.00	3.03	4.09	4.80	7.67	5.11	8.18	8.00
7+	1636.20	0.00	1.00	1.00	0.52	1.00	0.00	1.00	0.00

S Peptide 1-19									
ion	m/z	3V	8V	10V	12V	15V	20V	30V	50V
1+D	4189.30	0.00	0.00	0.00	0.00	0.00	0.00	0.00	0.00
1+	2095.15	0.00	0.00	0.00	0.00	0.00	0.00	0.00	0.00
2+	1048.07	0.00	4.89	14.80	31.40	70.20	133.00	162.00	187.00
3+	699.05	0.00	1.14	1.25	1.00	1.20	1.09	1.43	0.00
4+	524.53	0.00	0.00	0.00	0.00	0.00	0.00	0.00	0.00

S Peptide 1-20									
ion	m/z	3V	8V	10V	12V	15V	20V	30V	50V
1+D	4330.36	0.00	0.00	0.00	0.00	0.00	0.00	0.00	0.00
1+	2166.18	0.00	0.00	0.00	0.00	0.00	0.00	0.00	0.00
2+	1083.60	0.00	1.13	4.40	10.00	25.20	45.30	65.90	71.30
3+	722.72	0.00	0.60	0.00	0.00	0.00	0.00	0.00	0.00
4+	542.30	0.00	0.00	0.00	0.00	0.00	0.00	0.00	0.00

D=Dimer

Supplemental Table 3: continued

3rd determination

RNAse S Complex 1-19...21-124									
ion	m/z	3V	8V	10V	12V	15V	20V	30V	50V
5+	2727.08	0.00	n.d	0.00	n.d	n.d	0.00	0.00	0.00
6+	2272.78	5.74	n.d	8.57	n.d	n.d	9.74	8.66	5.89
7+	1948.17	75.00	n.d	99.70	n.d	n.d	21.50	21.60	11.80
8+	1704.79	5.81	n.d	6.40	n.d	n.d	1.05	2.00	0.50
9+	1515.49	0.00	n.d	0.00	n.d	n.d	0.00	0.00	0.00

RNAse S Complex 1-20...22-124									
ion	m/z	3V	8V	10V	12V	15V	20V	30V	50V
5+	2709.58	0.00	n.d	0.00	n.d	n.d	0.00	0.00	0.00
6+	2258.15	3.52	n.d	4.31	n.d	n.d	4.26	3.29	0.00
7+	1935.70	45.90	n.d	56.70	n.d	n.d	13.00	12.00	6.14
8+	1693.85	2.74	n.d	4.10	n.d	n.d	1.00	1.00	0.00
9+	1505.76	0.00	n.d	0.00	n.d	n.d	0.00	0.00	0.00

S Protein 21-124									
ion	m/z	3V	8V	10V	12V	15V	20V	30V	50V
3+	3845.75	0.00	n.d	0.00	n.d	n.d	0.00	0.00	0.00
4+	2884.67	0.00	n.d	0.00	n.d	n.d	0.00	3.98	5.38
5+	2307.79	0.00	n.d	13.10	n.d	n.d	77.70	112.00	120.00
6+	1923.36	0.00	n.d	8.33	n.d	n.d	8.23	14.00	16.40
7+	1648.75	0.00	n.d	1.00	n.d	n.d	0.50	0.00	1.00

S Protein 22-124									
ion	m/z	3V	8V	10V	12V	15V	20V	30V	50V
3+	3816.46	0.00	n.d	0.00	n.d	n.d	0.00	0.00	0.00
4+	2862.59	0.00	n.d	0.00	n.d	n.d	0.00	2.15	3.17
5+	2290.29	0.00	n.d	9.74	n.d	n.d	49.60	69.30	79.50
6+	1908.72	0.00	n.d	3.50	n.d	n.d	4.50	8.57	8.79
7+	1636.20	0.00	n.d	1.00	n.d	n.d	0.50	0.00	0.00

S Peptide 1-19									
ion	m/z	3V	8V	10V	12V	15V	20V	30V	50V
1+D	4189.30	0.00	n.d	0.00	n.d	n.d	0.00	0.00	0.00
1+	2095.15	0.00	n.d	0.00	n.d	n.d	0.00	0.00	0.00
2+	1048.07	0.00	n.d	16.10	n.d	n.d	136.00	160.00	187.00
3+	699.05	0.00	n.d	1.14	n.d	n.d	1.28	0.00	1.00
4+	524.53	0.00	n.d	0.00	n.d	n.d	0.00	0.00	0.00

S Peptide 1-20									
ion	m/z	3V	8V	10V	12V	15V	20V	30V	50V
1+D	4330.36	0.00	n.d	0.00	n.d	n.d	0.00	0.00	0.00
1+	2166.18	0.00	n.d	0.00	n.d	n.d	0.00	0.00	0.00
2+	1083.60	0.00	n.d	5.51	n.d	n.d	47.90	65.00	74.70
3+	722.72	0.00	n.d	0.00	n.d	n.d	0.00	0.00	0.00
4+	542.30	0.00	n.d	0.00	n.d	n.d	0.00	0.00	0.00

D=Dimer

Supplemental Table 4: Apex heights and mean charge states of educt and product ion signals upon gas phase dissociation of RNAse S dissolved in 5% MeOH / 200 mM ammonium acetate (pH 7).

1st determination [a)]

**ΔCV**	RNAse S				S-Protein				S-Peptide			
**[V]**	1-19..21-124		1-20..22-124		21-124		22-124		1-19		1-20	
	$m_{(1)}+$	$h_{(1)}$	$m_{(2)}+$	$h_{(2)}$	$p_{(1)}+$	$j_{(1)}$	$p_{(2)}+$	$j_{(2)}$	$n_{(1)}+$	$i_{(1)}$	$n_{(2)}+$	$i_{(2)}$
**3**	7	56.25	7.0	29.18	5.7	0.00	5.4	0.00	2.2	0.00	2.2	0.00
**8**	7	83.36	7.0	45.67	5.5	6.63	5.3	3.30	2.1	4.96	2.1	1.58
**10**	7	84.85	7.0	48.97	5.3	11.44	5.3	7.60	2.0	12.28	2.0	3.42
**12**	7	84.98	7.0	47.59	5.3	22.34	5.2	15.95	2.0	26.01	2.0	9.24
**15**	6.9	59.71	6.9	31.79	5.2	45.20	5.1	32.10	2.0	64.49	2.0	20.16
**20**	6.7	18.70	6.8	11.31	5.1	69.08	5.1	45.41	2.0	107.39	2.0	42.30
**30**	6.8	21.75	6.8	11.83	5.1	110.01	5.1	67.94	2.0	181.39	2.0	65.42
**50**	6.8	15.20	6.9	8.66	5.1	128.69	5.1	75.63	2.0	196.47	2.0	80.67

[a)] arbitrary units; cf. Supplemental Figure 1

2nd determination [a)]

**ΔCV**	RNAse S				S-Protein				S-Peptide			
**[V]**	1-19..21-124		1-20..22-124		21-124		22-124		1-19		1-20	
	$m_{(1)}+$	$h_{(1)}$	$m_{(2)}+$	$h_{(2)}$	$p_{(1)}+$	$j_{(1)}$	$p_{(2)}+$	$j_{(2)}$	$n_{(1)}+$	$i_{(1)}$	$n_{(2)}+$	$i_{(2)}$
**3**	7.0	68.16	7.0	39.82	-	0.00	-	0.00	-	0.00	-	0.00
**8**	7.0	95.01	7.0	56.74	5.6	7.09	5.7	3.46	2.2	4.88	2.4	1.12
**10**	7.0	99.47	7.0	55.14	5.5	11.31	5.4	10.03	2.1	14.86	2.0	4.43
**12**	7.0	89.81	7.0	53.23	5.3	27.91	5.3	18.27	2.0	31.78	2.0	10.06
**15**	6.9	60.19	6.9	33.77	5.2	60.84	5.2	37.89	2.0	71.21	2.0	25.25
**20**	6.8	23.01	6.8	12.24	5.1	109.41	5.0	50.53	2.0	133.70	2.0	45.30
**30**	6.8	20.73	6.9	13.19	5.1	112.34	5.1	71.81	2.0	162.84	2.0	65.90
**50**	6.7	11.40	6.8	6.75	5.1	120.06	5.1	73.74	2.0	187.13	2.0	71.30

[a)] arbitrary units; cf. Supplemental Figure 1

3rd determination [a)]

**ΔCV**	RNAse S				S-Protein				S-Peptide			
**[V]**	1-19..21-124		1-20..22-124		21-124		22-124		1-19		1-20	
	$m_{(1)}+$	$h_{(1)}$	$m_{(2)}+$	$h_{(2)}$	$p_{(1)}+$	$j_{(1)}$	$p_{(2)}+$	$j_{(2)}$	$n_{(1)}+$	$i_{(1)}$	$n_{(2)}+$	$i_{(2)}$
**3**	7.0	74.38	7.0	46.23	-	0.00	-	0.00	-	0.00	-	0.00
**8**	n.a.	n.a.	n.a.	n.a.	n.a.	n.a.	n.a.	n.a.	n.a.	n.a.	n.a.	n.a.
**10**	7.0	99.45	7.0	57.08	5.5	13.18	5.4	9.58	2.1	16.25	2.0	5.63
**12**	n.a.	n.a.	n.a.	n.a.	n.a.	n.a.	n.a.	n.a.	n.a.	n.a.	n.a.	n.a.
**15**	n.a.	n.a.	n.a.	n.a.	n.a.	n.a.	n.a.	n.a.	n.a.	n.a.	n.a.	n.a.
**20**	6.7	21.79	6.8	13.00	5.1	113.42	5.1	49.99	2.0	136.70	2.0	48.23
**30**	6.8	21.40	6.9	12.07	5.1	119.06	5.1	69.37	2.0	160.10	2.0	65.13
**50**	6.7	11.82	7.0	6.16	5.1	57.37	5.1	79.89	2.0	190.46	2.0	74.86

[a)] arbitrary units; cf. Supplemental Figure 1

Supplemental Table 5: Ion intensities, charge states, and m/z values for RNAse S at various collision cell voltage difference settings in 10% methanol/200 mM ammonium acetate (pH 7).

1st determination

RNAse S Complex 1-19...21-124

ion	m/z	3V	8V	11V	13V	15V	17V	20V	30V	50V
5+	2727.90	1.83	1.01	2.84	3.63	4.82	3.14	3.89	3.44	1.46
6+	2272.97	51.10	36.30	67.40	109.00	124.00	99.20	98.20	49.80	19.10
7+	1948.36	150.00	116.00	166.00	135.00	98.60	46.30	32.10	20.50	9.56
8+	1704.93	0.90	0.58	0.67	0.00	0.29	0.00	0.28	0.16	0.28
9+	1515.49	0.00	0.00	0.00	0.00	0.00	0.00	0.00	0.00	0.12

RNAse S Complex 1-20...22-124

ion	m/z	3V	8V	11V	13V	15V	17V	20V	30V	50V
5+	2709.68	0.00	0.00	0.00	0.93	0.80	1.41	2.31	1.84	0.86
6+	2258.30	31.20	20.80	0.00	67.30	71.80	55.60	57.00	22.90	4.22
7+	1935.95	78.10	64.70	48.30	70.50	49.90	23.20	20.30	12.70	5.69
8+	1693.60	0.56	0.46	0.18	0.00	0.00	0.11	0.30	0.00	0.00
9+	1505.78	0.00	0.00	0.16	0.00	0.00	0.00	0.00	0.00	0.00

S Protein 21-124

ion	m/z	3V	8V	11V	13V	15V	17V	20V	30V	50V
3+	3845.75	0.00	0.00	0.00	0.00	0.00	0.00	0.00	0.00	0.00
4+	2884.76	0.00	0.00	0.06	0.00	0.00	0.36	1.24	42.80	44.80
5+	2308.44	0.00	5.77	53.80	178.00	294.00	270.00	303.00	355.00	392.00
6+	1923.71	6.00	5.26	8.56	7.47	8.35	5.36	6.54	6.41	11.30
7+	1648.71	0.00	0.00	1.03	0.00	0.20	0.13	0.13	0.21	0.16

S Protein 22-124

ion	m/z	3V	8V	11V	13V	15V	17V	20V	30V	50V
3+	3816.46	0.00	0.00	0.00	0.00	0.00	0.00	0.00	0.00	0.00
4+	2862.45	0.00	0.00	0.00	0.00	0.00	0.40	1.10	29.20	28.40
5+	2289.00	0.00	12.80	42.80	120.00	190.00	175.00	194.00	217.00	241.00
6+	1908.50	1.06	1.00	2.43	3.15	4.09	3.04	2.62	1.01	5.71
7+	1636.20	0.00	0.00	0.00	0.00	0.00	0.00	0.17	0.06	0.16

S Peptide 1-19

ion	m/z	3V	8V	11V	13V	15V	17V	20V	30V	50V
1+D	4189.30	0.00	0.00	0.00	0.00	0.00	0.00	0.00	0.00	0.00
1+	2095.35	0.00	0.00	0.00	0.00	0.00	0.00	0.00	2.52	5.61
2+	1048.18	0.18	1.53	39.30	153.00	258.00	261.00	257.00	377.00	430.00
3+	699.15	0.00	1.00	0.00	0.09	0.34	0.21	0.33	0.68	0.25
4+	524.53	0.00	0.00	0.00	0.00	0.00	0.00	0.00	0.00	0.00

S Peptide 1-20

ion	m/z	3V	8V	11V	13V	15V	17V	20V	30V	50V
1+D	4330.36	0.00	0.00	0.00	0.00	0.00	0.00	0.00	0.00	0.00
1+	2166.18	0.00	0.00	0.00	0.00	0.00	0.00	0.00	1.19	1.05
2+	1083.57	0.06	0.34	9.50	42.40	78.90	83.00	95.90	141.00	157.00
3+	722.89	0.00	0.00	0.00	0.00	0.22	0.19	0.07	0.00	0.06
4+	542.30	0.00	0.00	0.00	0.00	0.00	0.00	0.00	0.00	0.00

D=Dimer

Supplemental Table 5: continued

2nd determination

RNAse S Complex 1-19...21-124										
ion	m/z	3V	8V	11V	13V	15V	17V	20V	30V	50V
5+	2727.22	0.46	0.70	1.06	0.75	0.94	1.44	2.11	1.31	1.12
6+	2272.67	108.00	134.00	153.00	114.00	122.00	115.00	177.00	54.10	22.00
7+	1948.17	177.00	213.00	161.00	71.30	41.90	28.20	35.80	13.30	7.51
8+	1705.04	1.29	1.05	0.57	0.59	0.42	0.00	0.25	0.09	0.17
9+	1515.49	0.00	0.00	0.00	0.00	0.00	0.00	0.00	0.00	0.00

RNAse S Complex 1-20...22-124										
ion	m/z	3V	8V	11V	13V	15V	17V	20V	30V	50V
5+	2709.58	0.26	0.36	0.87	0.46	1.22	1.40	1.51	1.50	0.70
6+	2257.97	57.80	75.20	87.50	64.80	68.30	64.00	97.50	26.40	8.64
7+	1935.71	96.20	111.00	86.70	39.70	21.90	15.70	20.40	7.22	4.65
8+	1693.89	0.68	0.68	0.50	0.13	0.35	0.19	0.27	0.22	0.20
9+	1505.76	0.00	0.00	0.00	0.00	0.00	0.00	0.00	0.00	0.15

S Protein 21-124										
ion	m/z	3V	8V	11V	13V	15V	17V	20V	30V	50V
3+	3845.75	0.00	0.00	0.00	0.00	0.00	0.00	0.00	0.00	0.00
4+	2884.67	0.00	0.00	0.00	0.00	0.00	1.01	2.20	39.40	46.00
5+	2308.56	24.80	32.00	78.90	121.00	151.00	151.00	250.00	188.00	185.00
6+	1923.25	8.19	9.08	8.22	4.29	3.32	2.57	3.49	4.23	3.74
7+	1648.75	0.00	0.00	0.00	0.00	0.23	0.00	0.00	0.00	0.22

S Protein 22-124										
ion	m/z	3V	8V	11V	13V	15V	17V	20V	30V	50V
3+	3816.46	0.00	0.00	0.00	0.00	0.00	0.00	0.00	0.00	0.00
4+	2862.59	0.00	0.25	0.00	0.00	0.00	0.71	2.11	24.60	27.90
5+	2288.33	51.00	64.20	76.60	95.80	104.00	108.00	171.00	125.00	115.00
6+	1908.23	1.70	2.37	1.93	1.26	1.30	1.36	1.53	2.23	2.57
7+	1636.20	0.00	0.18	0.00	0.00	0.00	0.32	0.00	0.00	0.18

S Peptide 1-19										
ion	m/z	3V	8V	11V	13V	15V	17V	20V	30V	50V
1+D	4189.30	0.00	0.00	0.00	0.00	0.00	0.00	0.00	0.00	0.00
1+	2095.15	0.00	0.00	0.00	0.00	0.00	0.00	0.00	4.19	7.46
2+	1048.59	0.29	7.58	80.00	142.00	189.00	195.00	281.00	379.00	405.00
3+	699.05	0.06	0.00	0.00	0.00	0.11	0.17	0.19	0.19	0.00
4+	524.53	0.00	0.00	0.00	0.00	0.00	0.00	0.00	0.00	0.00

S Peptide 1-20										
ion	m/z	3V	8V	11V	13V	15V	17V	20V	30V	50V
1+D	4330.36	0.00	0.00	0.00	0.00	0.00	0.00	0.00	0.00	0.00
1+	2166.18	0.00	0.00	0.00	0.00	0.00	0.00	0.00	0.00	2.28
2+	1083.59	0.18	1.67	21.40	39.40	64.90	71.40	107.00	145.00	157.00
3+	722.72	0.00	0.00	0.12	0.00	0.00	0.17	0.00	0.00	0.00
4+	542.30	0.00	0.00	0.00	0.00	0.00	0.00	0.00	0.00	0.00

D=Dimer

Supplemental Table 5: continued

3rd determination

RNAse S Complex 1-19...21-124										
ion	m/z	3V	8V	11V	13V	15V	17V	20V	30V	50V
5+	2727.59	1.59	1.57	2.88	1.07	3.15	2.84	3.68	1.96	0.59
6+	2272.93	130.00	183.00	234.00	195.00	229.00	178.00	269.00	52.70	14.60
7+	1948.41	215.00	281.00	278.00	127.00	84.00	50.40	60.30	15.70	5.57
8+	1705.16	1.03	0.39	0.31	0.30	0.33	0.38	0.75	0.37	0.29
9+	1515.49	0.00	0.00	0.06	0.00	0.13	0.15	0.00	0.12	0.18

RNAse S Complex 1-20...22-124										
ion	m/z	3V	8V	11V	13V	15V	17V	20V	30V	50V
5+	2709.68	0.62	0.58	0.65	1.56	1.54	1.70	2.75	1.64	0.34
6+	2258.39	79.60	109.00	137.00	116.00	135.00	108.00	153.00	25.80	6.34
7+	1935.92	119.00	161.00	155.00	69.9	44.50	27.10	33.30	8.68	4.14
8+	1694.24	0.77	0.69	0.28	0.38	0.28	0.13	0.39	0.41	0.26
9+	1505.76	0.00	0.00	0.00	0.00	0.00	0.07	0.00	0.11	0.18

S Protein 21-124										
ion	m/z	3V	8V	11V	13V	15V	17V	20V	30V	50V
3+	3845.75	0.00	0.00	0.00	0.00	0.00	0.00	0.00	0.00	0.00
4+	2884.90	0.22	0.12	0.00	0.38	0.71	0.94	3.58	40.50	33.60
5+	2308.05	10.40	15.50	92.40	184.00	288.00	259.00	429.00	228.00	156.00
6+	1923.58	8.76	12.60	11.20	6.18	5.21	4.20	6.46	3.63	3.94
7+	1648.87	0.22	0.00	0.00	0.24	0.12	0.24	0.57	0.33	0.12

S Protein 22-124										
ion	m/z	3V	8V	11V	13V	15V	17V	20V	30V	50V
3+	3816.46	0.00	0.00	0.00	0.00	0.00	0.00	0.00	0.00	0.00
4+	2862.33	0.21	0.30	0.00	0.35	0.73	0.87	2.65	26.80	22.50
5+	2288.35	34.90	39.80	75.80	125.00	189.00	174.00	278.00	140.00	98.90
6+	1908.24	1.03	1.67	1.72	1.21	2.01	1.84	2.82	2.38	1.88
7+	1636.20	0.00	0.06	0.00	0.18	0.13	0.00	0.21	0.20	0.00

S Peptide 1-19										
ion	m/z	3V	8V	11V	13V	15V	17V	20V	30V	50V
1+D	4189.30	0.00	0.00	0.00	0.00	0.00	0.00	0.00	0.00	0.00
1+	2095.15	0.00	0.00	0.00	0.00	0.00	0.00	4.36	3.58	5.92
2+	1048.07	0.21	6.41	71.10	160.00	253.00	270.00	493.00	372.00	362.00
3+	699.05	0.00	0.00	0.06	0.00	0.00	0.31	0.48	0.00	0.00
4+	524.53	0.00	0.00	0.00	0.00	0.00	0.00	0.00	0.00	0.00

S Peptide 1-20										
ion	m/z	3V	8V	11V	13V	15V	17V	20V	30V	50V
1+D	4330.36	0.00	0.00	0.00	0.00	0.00	0.00	0.00	0.00	0.00
1+	2166.18	0.00	0.00	0.00	0.00	0.00	0.00	0.00	0.00	2.14
2+	1083.60	0.00	1.04	18.40	47.20	90.50	100.00	190.00	144.00	141.00
3+	722.72	0.00	0.00	0.00	0.00	0.00	0.00	0.24	0.00	0.23
4+	542.30	0.00	0.00	0.00	0.00	0.00	0.00	0.00	0.00	0.00

D=Dimer

Supplemental Table 6: Apex heights and mean charge states of educt and product ion signals upon gas phase dissociation of RNAse S dissolved in 10 % MeOH / 200 mM ammonium acetate (pH 7).

1st determination [a)]

**ΔCV**	RNAse S				S-Protein				S-Peptide			
**[V]**	1-19..21-124		1-20..22-124		21-124		22-124		1-19		1-20	
	$m_{(1)}$+	$h_{(1)}$	$m_{(2)}$+	$h_{(2)}$	$p_{(1)}$+	$j_{(1)}$	$p_{(2)}$+	$j_{(2)}$	$n_{(1)}$+	$i_{(1)}$	$n_{(2)}$+	$i_{(2)}$
**3**	6.7	150.63	6.7	79.04	6.0	6.00	6.0	1.06	2.0	0.18	2.0	0.07
**8**	6.7	117.42	6.8	65.32	5.5	5.92	5.1	12.84	2.4	1.51	2.0	0.35
**11**	6.7	169.46	7.0	48.58	5.2	53.28	5.1	42.85	2.0	40.45	2.0	9.78
**13**	6.5	141.61	6.5	73.14	5.0	179.10	5.0	120.54	2.0	157.44	2.0	43.64
**15**	6.5	125.54	6.4	73.50	5.0	295.43	5.0	190.09	2.0	265.43	2.0	81.14
**17**	6.3	101.91	6.3	56.47	5.0	271.48	5.0	176.16	2.0	268.56	2.0	85.37
**20**	6.2	98.79	6.2	56.98	5.0	305.32	5.0	195.58	2.0	264.41	2.0	98.68
**30**	6.2	51.54	6.3	23.88	4.9	358.12	4.9	224.21	2.0	386.44	2.0	144.48
**50**	6.3	19.91	6.5	5.74	4.9	397.52	4.9	244.36	2.0	439.48	2.0	161.00

[a)] arbitrary units; cf. Figure 3

2nd determination [a)]

**ΔCV**	RNAse S				S-Protein				S-Peptide			
**[V]**	1-19..21-124		1-20..22-124		21-124		22-124		1-19		1-20	
	$m_{(1)}$+	$h_{(1)}$	$m_{(2)}$+	$h_{(2)}$	$p_{(1)}$+	$j_{(1)}$	$p_{(2)}$+	$j_{(2)}$	$n_{(1)}$+	$i_{(1)}$	$n_{(2)}$+	$i_{(2)}$
**3**	6.6	178.50	6.6	96.00	5.3	96.00	5.0	51.30	2.1	0.30	2.0	0.18
**8**	6.6	217.22	6.6	111.64	5.2	111.64	5.0	64.62	2.0	7.81	2.0	1.72
**11**	6.5	165.55	6.5	89.17	5.1	89.17	5.0	78.49	2.0	82.33	2.0	21.99
**13**	6.4	116.12	6.4	65.55	5.0	65.55	5.0	96.26	2.0	146.14	2.0	96.26
**15**	6.3	123.01	6.2	69.31	5.0	69.31	5.0	104.49	2.0	194.47	2.0	66.80
**17**	6.2	119.16	6.2	64.79	5.0	64.79	5.0	108.77	2.0	200.63	2.0	73.43
**20**	6.2	177.28	6.2	99.01	5.0	99.01	5.0	173.30	2.0	289.13	2.0	110.13
**30**	6.2	54.50	6.2	26.62	4.9	26.62	4.9	126.13	2.0	387.73	2.0	149.24
**50**	6.2	22.23	6.3	8.96	4.8	8.96	4.8	116.52	2.0	412.82	2.0	160.37

[a)] arbitrary units; cf. Figure 3

3rd determination [a)]

**ΔCV**	RNAse S				S-Protein				S-Peptide			
**[V]**	1-19..21-124		1-20..22-124		21-124		22-124		1-19		1-20	
	$m_{(1)}$+	$h_{(1)}$	$m_{(2)}$+	$h_{(2)}$	$p_{(1)}$+	$j_{(1)}$	$p_{(2)}$+	$j_{(2)}$	$n_{(1)}$+	$i_{(1)}$	$n_{(2)}$+	$i_{(2)}$
**3**	6.6	216.06	6.6	121.18	5.5	35.24	5.0	35.24	2.0	0.22	-	-
**8**	6.6	286.31	6.6	161.90	5.4	15.84	5.0	40.20	2.0	6.60	2.0	1.07
**11**	6.5	277.28	6.5	158.20	5.1	92.77	5.0	76.20	2.0	73.16	2.0	18.94
**13**	6.4	200.68	6.4	120.32	5.0	185.14	5.0	125.68	2.0	164.68	2.0	48.58
**15**	6.3	232.78	6.2	138.67	5.0	289.83	5.0	190.07	2.0	93.15	2.0	93.15
**17**	6.2	181.59	6.2	108.78	5.0	260.73	5.0	175.44	2.0	277.80	2.0	102.92
**20**	6.2	270.91	6.2	162.23	5.0	433.17	5.0	281.03	2.0	504.90	2.0	195.48
**30**	6.2	53.61	6.2	26.36	4.9	229.49	4.9	141.04	2.0	380.94	2.0	148.21
**50**	6.3	14.93	6.4	6.55	4.9	157.36	4.8	100.07	2.0	369.42	2.0	143.90

[a)] arbitrary units; cf. Figure 3

Supplemental Table 7: Ion intensities, charge states, and m/z values for RNAse S at various collision cell voltage difference settings in 20% methanol/200 mM ammonium acetate (pH 7).

1st determination

RNAse S Complex 1-19...21-124

ion	m/z	3V	8V	11V	13V	15V	17V	20V	30V	50V
5+	2727.01	1.44	1.89	2.84	2.79	2.33	3.31	2.39	1.85	0.56
6+	2272.76	87.40	116.00	137.00	240.00	157.00	140.00	90.40	52.30	16.00
7+	1948.23	199.00	244.00	259.00	227.00	102.00	62.00	31.70	13.90	8.19
8+	1704.76	0.47	0.48	0.58	0.36	0.12	0.13	0.00	0.00	0.00
9+	1515.49	0.00	0.00	0.00	0.00	0.00	0.00	0.00	0.00	0.00

RNAse S Complex 1-20...22-124

ion	m/z	3V	8V	11V	13V	15V	17V	20V	30V	50V
5+	2709.29	0.49	0.63	1.12	1.73	1.60	2.03	1.57	1.34	0.48
6+	2258.32	51.90	71.29	87.60	152.00	99.00	83.80	58.10	27.00	7.33
7+	1935.71	112.00	132.00	144.00	123.00	54.80	36.70	16.30	8.49	4.41
8+	1693.78	0.55	0.33	0.37	0.00	0.16	0.18	0.00	0.00	0.00
9+	1505.78	0.00	0.00	0.00	0.00	0.00	0.00	0.00	0.00	0.00

S Protein 21-124

ion	m/z	3V	8V	11V	13V	15V	17V	20V	30V	50V
3+	3845.75	0.00	0.00	0.00	0.00	0.00	0.00	0.00	0.00	0.00
4+	2884.76	0.00	0.00	0.00	0.00	0.00	1.21	1.35	29.80	38.20
5+	2308.47	4.39	8.12	37.10	142.00	172.00	189.00	144.00	155.00	152.00
6+	1923.41	9.08	13.00	14.30	14.90	8.92	6.54	3.81	4.61	5.57
7+	1648.71	0.29	0.37	0.37	0.30	0.12	0.16	0.00	0.00	0.00

S Protein 22-124

ion	m/z	3V	8V	11V	13V	15V	17V	20V	30V	50V
3+	3816.46	0.00	0.00	0.00	0.00	0.00	0.00	0.00	0.00	0.00
4+	2862.45	0.00	0.00	0.00	0.00	0.00	1.20	1.05	19.30	23.00
5+	2289.11	24.30	37.20	37.60	102.00	110.00	117.00	90.80	91.80	96.00
6+	1908.04	1.10	3.85	3.96	5.75	3.97	3.34	2.66	2.36	4.08
7+	1636.20	0.00	0.00	0.13	0.18	0.22	0.09	0.20	0.00	0.00

S Peptide 1-19

ion	m/z	3V	8V	11V	13V	15V	17V	20V	30V	50V
1+D	4189.30	0.00	0.00	0.00	0.00	0.00	0.00	0.00	0.00	0.00
1+	2095.35	0.00	0.00	0.00	0.00	0.00	0.00	0.00	1.19	3.36
2+	1048.18	0.00	0.79	23.10	100.00	124.00	153.00	131.00	162.00	194.00
3+	699.15	0.00	1.00	0.00	0.15	0.00	0.00	0.00	0.00	0.00
4+	524.53	0.00	0.00	0.00	0.00	0.00	0.00	0.00	0.00	0.00

S Peptide 1-20

ion	m/z	3V	8V	11V	13V	15V	17V	20V	30V	50V
1+D	4330.36	0.00	0.00	0.00	0.00	0.00	0.00	0.00	0.00	0.00
1+	2166.18	0.00	0.00	0.00	0.00	0.00	0.00	0.00	0.00	1.26
2+	1083.57	0.06	0.30	6.80	30.00	39.70	56.30	49.30	59.30	70.80
3+	722.89	0.00	0.00	0.00	0.00	0.00	0.00	0.00	0.00	0.00
4+	542.30	0.00	0.00	0.00	0.00	0.00	0.00	0.00	0.00	0.00

D=Dimer

Supplemental Table 7: continued

2nd determination

RNAse S Complex 1-19...21-124										
ion	m/z	3V	8V	11V	13V	15V	17V	20V	30V	50V
5+	2727.38	1.13	1.35	1.25	1.70	1.48	3.21	4.58	4.05	1.50
6+	2272.69	53.20	65.10	65.00	72.60	75.90	85.20	100.00	58.00	15.40
7+	1948.26	106.00	138.00	102.00	87.80	53.60	42.60	26.40	13.50	6.14
8+	1705.18	0.30	0.46	0.19	0.00	0.12	0.00	0.00	0.12	0.00
9+	1515.49	0.00	0.00	0.00	0.00	0.00	0.00	0.00	0.00	0.00

RNAse S Complex 1-20...22-124										
ion	m/z	3V	8V	11V	13V	15V	17V	20V	30V	50V
5+	2709.87	0.39	0.55	0.42	0.51	0.79	1.28	1.91	2.53	0.73
6+	2258.34	34.00	41.30	39.00	46.30	49.90	54.60	56.90	30.00	7.05
7+	1935.82	59.80	76.40	61.60	46.80	31.60	23.60	15.40	7.05	3.17
8+	1693.89	0.16	0.33	0.12	0.00	0.00	0.00	0.00	0.00	0.00
9+	1505.76	0.00	0.00	0.00	0.00	0.00	0.00	0.00	0.13	0.00

S Protein 21-124										
ion	m/z	3V	8V	11V	13V	15V	17V	20V	30V	50V
3+	3845.75	0.00	0.00	0.00	0.00	0.00	0.00	0.00	0.00	0.00
4+	2884.14	0.00	0.00	0.00	0.12	0.24	0.00	1.77	31.10	42.70
5+	2308.56	6.54	5.42	19.40	57.80	94.40	144.00	200.00	209.00	220.00
6+	1923.58	8.19	6.88	5.15	5.09	3.68	3.08	3.99	3.93	6.64
7+	1648.38	0.00	0.15	0.17	0.00	0.17	0.00	0.00	0.12	0.00

S Protein 22-124										
ion	m/z	3V	8V	11V	13V	15V	17V	20V	30V	50V
3+	3816.46	0.00	0.00	0.00	0.00	0.00	0.00	0.00	0.00	0.00
4+	2862.55	0.18	0.18	0.00	0.37	0.15	0.00	1.39	18.90	28.80
5+	2288.88	18.00	20.30	18.80	43.40	68.40	95.50	127.00	133.00	137.00
6+	1908.90	1.64	1.13	1.48	0.91	1.62	1.96	2.47	1.59	3.20
7+	1636.58	0.00	0.10	0.00	0.00	0.15	0.00	0.00	0.00	0.00

S Peptide 1-19										
ion	m/z	3V	8V	11V	13V	15V	17V	20V	30V	50V
1+D	4189.30	0.00	0.00	0.00	0.00	0.00	0.00	0.00	0.00	0.00
1+	2094.30	0.00	0.00	0.00	0.00	0.00	0.00	0.00	1.22	4.52
2+	1048.23	0.00	0.88	16.40	47.40	83.20	128.00	201.00	288.00	297.00
3+	699.17	0.00	0.00	0.00	0.00	0.00	0.00	0.19	0.18	0.12
4+	524.53	0.00	0.00	0.00	0.00	0.00	0.00	0.00	0.00	0.00

S Peptide 1-20										
ion	m/z	3V	8V	11V	13V	15V	17V	20V	30V	50V
1+D	4330.36	0.00	0.00	0.00	0.00	0.00	0.00	0.00	0.00	0.00
1+	2166.18	0.00	0.00	0.00	0.00	0.00	0.00	0.00	0.00	1.75
2+	1083.59	0.00	0.26	3.63	13.60	26.30	43.90	69.10	101.00	112.00
3+	722.72	0.00	0.00	0.00	0.00	0.00	0.00	0.00	0.00	0.00
4+	542.30	0.00	0.00	0.00	0.00	0.00	0.00	0.00	0.00	0.00

D=Dimer

Supplemental Table 8: Apex heights and mean charge states of educt and product ion signals upon gas phase dissociation of RNAse S dissolved in 20 % MeOH / 200 mM ammonium acetate (pH 7).

1st determination [a)]

**ΔCV**	RNAse S				S-Protein				S-Peptide			
**[V]**	1-19..21-124		1-20..22-124		21-124		22-124		1-19		1-20	
	$m_{(1)}+$	$h_{(1)}$	$m_{(2)}+$	$h_{(2)}$	$p_{(1)}+$	$j_{(1)}$	$p_{(2)}+$	$j_{(2)}$	$n_{(1)}+$	$i_{(1)}$	$n_{(2)}+$	$i_{(2)}$
**3**	6.7	221.25	5.7	125.77	5.7	9.36	5.0	24.46	-	0.00	2.0	0.07
**8**	6.7	264.91	5.6	139.15	5.6	13.31	5.1	37.51	2.6	1.12	2.0	0.31
**11**	6.6	272.56	5.3	157.98	5.3	37.71	5.1	37.82	2.0	23.78	2.0	7.01
**13**	6.5	250.85	5.1	165.98	8.2	142.24	7.7	102.57	2.0	103.06	2.0	30.93
**15**	6.4	181.11	5.0	108.60	5.1	173.04	5.0	110.49	2.0	127.85	2.0	40.93
**17**	6.3	157.53	5.0	87.32	5.0	190.80	5.0	118.41	2.0	157.75	2.0	58.05
**20**	6.2	108.03	5.0	62.37	5.0	145.72	5.0	91.87	2.0	135.07	2.0	50.83
**30**	6.2	55.79	4.9	28.18	4.9	174.06	4.9	94.21	2.0	167.08	2.0	61.14
**50**	6.3	17.27	4.8	7.77	4.8	153.94	4.8	101.16	2.0	200.15	2.0	73.05

[a)] arbitrary units; cf. Supplemental Figure 2

2nd determination [a)]

**ΔCV**	RNAse S				S-Protein				S-Peptide			
**[V]**	1-19..21-124		1-20..22-124		21-124		22-124		1-19		1-20	
	$m_{(1)}+$	$h_{(1)}$	$m_{(2)}+$	$h_{(2)}$	$p_{(1)}+$	$j_{(1)}$	$p_{(2)}+$	$j_{(2)}$	$n_{(1)}+$	$i_{(1)}$	$n_{(2)}+$	$i_{(2)}$
**3**	6.7	109.76	6.6	60.58	5.6	8.30	5.1	18.30	-	0.00	-	0.00
**8**	6.7	140.41	6.6	77.54	5.6	7.00	5.1	20.59	2.0	0.91	2.0	0.27
**11**	6.6	103.97	6.6	62.81	5.2	19.52	5.1	18.91	2.0	16.91	2.0	3.74
**13**	6.5	100.54	6.5	49.43	5.1	58.28	5.0	43.93	2.0	48.87	2.0	14.02
**15**	6.4	79.86	6.4	51.71	5.0	94.96	5.0	68.72	2.0	85.78	2.0	27.12
**17**	6.3	87.10	6.3	54.75	5.0	144.13	5.0	95.62	2.0	131.97	2.0	45.26
**20**	6.2	99.55	6.2	56.54	5.0	202.43	5.0	128.88	2.0	207.17	2.0	71.25
**30**	6.1	57.92	6.1	30.04	4.9	210.40	4.9	133.62	2.0	296.92	2.0	104.14
**50**	6.2	15.87	6.2	7.40	4.9	221.31	4.9	138.42	2.0	306.34	2.0	115.54

[a)] arbitrary units; cf. Supplemental Figure 2

Supplemental Table 9: Ion intensities, charge states, and m/z values for RNAse S at various collision cell voltage difference settings in 30% methanol/200 mM ammonium acetate (pH 7).

1st determination

RNAse S Complex 1-19...21-124

ion	m/z	3V	8V	11V	13V	15V	17V	20V	30V	50V
5+	2727.40	2.22	4.08	5.00	6.43	6.79	9.01	12.20	13.10	3.49
6+	2272.58	84.80	164.00	215.00	248.00	282.00	311.00	263.00	152.00	51.80
7+	1948.08	165.00	291.00	306.00	272.00	217.00	159.00	124.00	74.70	35.10
8+	1704.48	0.39	0.59	0.40	0.51	0.40	0.41	0.00	0.47	0.00
9+	1515.49	0.00	0.00	0.00	0.00	0.00	0.00	0.00	0.00	0.00

RNAse S Complex 1-20...22-124

ion	m/z	3V	8V	11V	13V	15V	17V	20V	30V	50V
5+	2709.37	0.51	1.07	1.44	1.89	2.32	4.12	7.84	6.55	2.52
6+	2258.23	50.60	99.60	132.00	153.00	168.00	194.00	161.00	84.50	22.10
7+	1935.66	87.50	155.00	165.00	146.00	114.00	87.70	71.00	45.50	19.30
8+	1693.96	0.39	0.36	0.47	0.00	0.38	0.35	0.29	0.41	0.00
9+	1505.78	0.14	0.00	0.00	0.00	0.18	0.00	0.00	0.00	0.00

S Protein 21-124

ion	m/z	3V	8V	11V	13V	15V	17V	20V	30V	50V
3+	3845.75	0.00	0.00	0.00	0.00	0.00	0.00	0.00	0.00	0.00
4+	2884.76	0.00	0.00	0.00	0.00	0.00	2.05	4.25	66.50	97.20
5+	2307.18	23.60	26.30	82.90	209.00	363.00	470.00	527.00	699.00	648.00
6+	1923.44	9.48	16.90	20.40	23.90	28.00	28.60	27.70	33.80	29.90
7+	1948.95	0.00	0.22	0.45	0.33	0.46	0.64	0.79	0.83	0.75

S Protein 22-124

ion	m/z	3V	8V	11V	13V	15V	17V	20V	30V	50V
3+	3816.46	0.00	0.00	0.00	0.00	0.00	0.00	0.00	0.00	0.00
4+	2862.71	0.00	0.00	0.00	0.00	0.00	0.00	3.89	47.90	66.90
5+	2288.03	0.00	59.50	74.40	154.00	250.00	323.00	349.00	443.00	412.00
6+	1908.07	1.06	4.07	7.35	10.80	14.00	15.70	15.30	18.70	19.70
7+	1636.34	0.00	0.24	0.36	0.27	0.50	0.36	0.54	0.37	0.36

S Peptide 1-19

ion	m/z	3V	8V	11V	13V	15V	17V	20V	30V	50V
1+D	4189.30	0.00	0.00	0.00	0.00	0.00	0.00	0.00	0.00	0.00
1+	2095.01	0.00	0.00	0.00	0.00	0.00	0.00	0.00	7.89	13.30
2+	1048.10	1.97	3.75	52.50	152.00	283.00	382.00	467.00	904.00	998.00
3+	699.15	0.00	0.00	0.00	0.14	0.22	0.47	0.55	1.84	1.52
4+	524.53	0.00	0.00	0.00	0.00	0.00	0.00	0.00	0.00	0.00

S Peptide 1-20

ion	m/z	3V	8V	11V	13V	15V	17V	20V	30V	50V
1+D	4330.36	0.00	0.00	0.00	0.00	0.00	0.00	0.00	0.00	0.00
1+	2166.11	0.00	0.00	0.00	0.00	0.00	0.00	0.00	0.00	1.46
2+	1083.63	0.51	0.89	12.60	39.70	85.70	124.00	160.00	313.00	357.00
3+	722.78	0.00	0.00	0.00	0.00	0.00	0.19	0.23	0.56	0.23
4+	542.30	0.00	0.00	0.00	0.00	0.00	0.00	0.00	0.00	0.00

D=Dimer

Supplemental Table 9: continued

2nd determination

RNAse S Complex 1-19...21-124										
ion	m/z	3V	8V	11V	13V	15V	17V	20V	30V	50V
5+	2727.55	4.90	5.94	8.56	10.50	10.40	11.20	10.10	8.86	3.31
6+	2272.83	114.00	147.00	190.00	200.00	200.00	234.00	215.00	119.00	44.50
7+	1948.23	321.00	413.00	420.00	307.00	198.00	141.00	90.40	44.00	23.50
8+	1704.86	2.23	1.51	1.09	0.76	0.39	0.62	0.52	0.00	0.00
9+	1515.49	0.00	0.00	0.00	0.00	0.00	0.00	0.00	0.00	0.00

RNAse S Complex 1-20...22-124										
ion	m/z	3V	8V	11V	13V	15V	17V	20V	30V	50V
5+	2709.54	1.51	2.25	3.11	4.21	5.44	6.12	5.43	4.82	2.40
6+	2258.22	73.80	94.00	124.00	127.00	127.00	137.00	134.00	62.10	16.80
7+	1935.71	177.00	231.00	228.00	164.00	105.00	74.90	46.80	25.50	14.50
8+	1693.84	0.84	1.41	0.52	0.40	0.25	0.30	0.25	0.00	0.24
9+	1505.76	0.00	0.00	0.00	0.00	0.00	0.00	0.00	0.00	0.00

S Protein 21-124										
ion	m/z	3V	8V	11V	13V	15V	17V	20V	30V	50V
3+	3845.75	0.00	0.00	0.00	0.00	0.00	0.00	0.00	0.00	0.00
4+	2884.74	0.00	0.00	0.00	0.00	0.00	0.00	0.00	61.20	92.90
5+	2308.72	18.20	19.20	89.80	198.00	316.00	445.00	456.00	511.00	610.00
6+	1923.36	23.00	27.80	29.30	28.20	23.60	22.40	18.60	21.70	25.50
7+	1648.57	0.76	0.68	0.92	0.71	0.67	0.61	0.40	0.63	0.43

S Protein 22-124										
ion	m/z	3V	8V	11V	13V	15V	17V	20V	30V	50V
3+	3816.46	0.00	0.00	0.00	0.00	0.00	0.00	0.00	0.00	0.00
4+	2862.98	0.00	0.00	0.00	0.00	0.00	0.00	0.00	39.90	62.20
5+	2288.30	48.90	52.90	67.00	144.00	212.00	292.00	296.00	318.00	370.00
6+	1908.98	8.83	10.50	12.20	11.10	11.70	12.10	11.60	13.30	2.88
7+	1636.46	0.54	0.65	0.73	0.35	0.58	0.60	0.55	0.35	0.41

S Peptide 1-19										
ion	m/z	3V	8V	11V	13V	15V	17V	20V	30V	50V
1+D	4189.30	0.00	0.00	0.00	0.00	0.00	0.00	0.00	0.00	0.00
1+	2095.27	0.00	0.00	0.00	0.00	0.00	0.00	0.00	6.06	14.80
2+	1048.14	4.72	10.90	62.90	159.00	282.00	440.00	528.00	800.00	1004.00
3+	699.15	0.00	0.18	0.14	0.12	0.47	0.65	0.85	1.16	1.21
4+	524.53	0.00	0.00	0.00	0.00	0.00	0.00	0.00	0.00	0.00

S Peptide 1-20										
ion	m/z	3V	8V	11V	13V	15V	17V	20V	30V	50V
1+D	4330.36	0.00	0.00	0.00	0.00	0.00	0.00	0.00	0.00	0.00
1+	2166.39	0.00	0.00	0.00	0.00	0.00	0.00	0.00	0.00	4.07
2+	1083.69	1.13	3.08	14.70	40.10	83.90	137.00	176.00	272.00	372.00
3+	722.86	0.00	0.00	0.00	0.00	0.22	0.25	0.27	0.20	0.22
4+	542.30	0.00	0.00	0.00	0.00	0.00	0.00	0.00	0.00	0.00

D=Dimer

Supplemental Table 9: continued

3rd determination

RNAse S Complex 1-19...21-124										
ion	m/z	3V	8V	11V	13V	15V	17V	20V	30V	50V
5+	2727.40	3.54	5.12	7.61	8.49	9.88	10.20	10.70	9.34	3.20
6+	2272.84	108.00	150.00	236.00	186.00	187.00	203.00	206.00	117.00	40.00
7+	1948.44	249.00	337.00	439.00	258.00	177.00	132.00	84.90	45.60	21.80
8+	1705.10	1.23	1.11	0.95	0.89	0.00	0.65	0.42	0.00	0.00
9+	1515.49	0.00	0.00	0.00	0.00	0.00	0.00	0.00	0.00	0.00

RNAse S Complex 1-20...22-124										
ion	m/z	3V	8V	11V	13V	15V	17V	20V	30V	50V
5+	2709.68	0.98	1.84	3.25	3.91	4.62	5.93	5.22	6.08	2.33
6+	2258.32	67.70	90.70	146.00	121.00	112.00	125.00	126.00	63.20	17.90
7+	1935.89	137.00	185.00	241.00	143.00	93.80	65.00	43.10	24.10	11.80
8+	1694.43	0.63	0.44	0.64	0.42	0.00	0.00	0.00	0.00	0.00
9+	1505.76	0.00	0.00	0.00	0.00	0.00	0.00	0.00	0.00	0.00

S Protein 21-124										
ion	m/z	3V	8V	11V	13V	15V	17V	20V	30V	50V
3+	3845.75	0.00	0.00	0.00	0.00	0.00	0.00	0.00	0.00	0.00
4+	2884.96	0.00	0.00	0.00	0.00	0.00	0.00	4.26	60.10	92.00
5+	2308.10	21.70	23.90	102.00	177.00	270.00	382.00	442.00	529.00	582.00
6+	1923.63	15.60	21.50	30.30	22.10	20.70	17.70	18.10	21.20	22.80
7+	1648.81	0.34	0.43	0.88	0.78	0.81	0.62	0.57	0.00	0.00

S Protein 22-124										
ion	m/z	3V	8V	11V	13V	15V	17V	20V	30V	50V
3+	3816.46	0.00	0.00	0.00	0.00	0.00	0.00	0.00	0.00	0.00
4+	2863.10	0.00	0.00	0.00	0.00	0.00	0.00	4.86	42.70	56.40
5+	2290.71	46.30	61.50	89.10	134.00	185.00	254.00	298.00	338.00	356.00
6+	1908.95	4.51	7.99	10.70	11.50	9.96	9.65	9.35	11.90	14.10
7+	1636.52	0.36	0.00	0.00	0.34	0.53	0.39	0.47	0.00	0.00

S Peptide 1-19										
ion	m/z	3V	8V	11V	13V	15V	17V	20V	30V	50V
1+D	4189.30	0.00	0.00	0.00	0.00	0.00	0.00	0.00	0.00	0.00
1+	2095.33	0.00	0.00	0.00	0.00	0.00	0.00	0.00	6.65	14.30
2+	1048.23	4.80	11.10	67.10	142.00	240.00	386.00	511.00	838.00	979.00
3+	699.21	0.21	0.24	0.37	0.38	0.24	0.66	1.02	1.06	1.57
4+	524.53	0.00	0.00	0.00	0.00	0.00	0.00	0.00	0.00	0.00

S Peptide 1-20										
ion	m/z	3V	8V	11V	13V	15V	17V	20V	30V	50V
1+D	4330.36	0.00	0.00	0.00	0.00	0.00	0.00	0.00	0.00	0.00
1+	2166.44	0.00	0.00	0.00	0.00	0.00	0.00	0.00	0.00	6.96
2+	1083.75	1.09	3.30	18.00	37.60	70.20	125.00	171.00	292.00	349.00
3+	722.72	0.00	0.00	0.19	0.34	0.00	0.00	0.00	0.44	0.53
4+	542.30	0.00	0.00	0.00	0.00	0.00	0.00	0.00	0.00	0.00

D=Dimer

Supplemental Table 10: Apex heights and mean charge states of educt and product ion signals upon gas phase dissociation of RNAse S dissolved in 30 % MeOH / 200 mM ammonium acetate (pH 7).

1st determination [a)]

ΔCV	RNAse S				S-Protein				S-Peptide			
[V]	1-19..21-124		1-20..22-124		21-124		22-124		1-19		1-20	
	$m_{(1)}+$	$h_{(1)}$	$m_{(2)}+$	$h_{(2)}$	$p_{(1)}+$	$j_{(1)}$	$p_{(2)}+$	$j_{(2)}$	$n_{(1)}+$	$i_{(1)}$	$n_{(2)}+$	$i_{(2)}$
**3**	6.7	168.80	6.6	89.17	5.3	23.57	6.0	1.07	2.0	1.98	2.0	0.51
**8**	6.6	295.32	6.6	158.57	5.4	26.24	5.1	60.15	2.0	3.76	2.0	0.91
**11**	6.6	316.08	6.6	166.74	5.2	83.72	5.1	75.73	2.0	52.77	2.0	12.86
**13**	6.5	278.09	6.5	154.81	5.1	217.14	5.1	157.25	2.0	152.81	2.0	40.53
**15**	6.4	284.37	6.4	174.31	5.1	368.86	5.1	253.44	2.0	284.11	2.0	87.50
**17**	6.3	313.70	6.3	199.40	5.1	473.62	5.0	328.33	2.0	383.53	2.0	126.52
**20**	6.3	273.23	6.3	165.09	5.0	532.22	5.0	354.71	2.0	469.52	2.0	163.25
**30**	6.3	156.78	6.3	86.26	5.0	707.61	4.9	440.30	2.0	913.93	2.0	319.39
**50**	6.4	53.27	6.3	22.31	4.9	655.79	4.9	416.78	2.0	1003.46	2.0	365.29

[a)] arbitrary units; cf. Supplemental Figure 3

2nd determination [a)]

ΔCV	RNAse S				S-Protein				S-Peptide			
[V]	1-19..21-124		1-20..22-124		21-124		22-124		1-19		1-20	
	$m_{(1)}+$	$h_{(1)}$	$m_{(2)}+$	$h_{(2)}$	$p_{(1)}+$	$j_{(1)}$	$p_{(2)}+$	$j_{(2)}$	$n_{(1)}+$	$i_{(1)}$	$n_{(2)}+$	$i_{(2)}$
**3**	6.7	321.66	6.7	177.22	5.6	23.44	5.2	49.08	2.0	4.74	2.0	1.15
**8**	6.7	413.87	6.7	232.36	5.6	28.37	5.2	53.13	2.0	10.98	2.0	3.15
**11**	6.7	426.98	6.6	229.66	5.3	90.54	5.2	67.24	2.0	63.22	2.0	15.01
**13**	6.6	307.60	6.5	167.14	5.1	198.20	5.1	143.90	2.0	159.67	2.0	40.92
**15**	6.5	203.91	6.4	128.25	5.1	315.65	5.1	212.95	2.0	283.38	2.0	85.65
**17**	6.3	237.86	6.3	138.55	5.0	444.31	5.0	293.00	2.0	442.54	2.0	137.40
**20**	6.3	214.79	6.2	135.40	5.0	455.22	5.0	296.96	2.0	530.57	2.0	176.41
**30**	6.2	118.66	6.2	62.02	4.9	515.20	4.9	322.96	2.0	808.35	2.0	277.44
**50**	6.3	45.37	6.4	16.98	4.9	618.70	4.9	374.96	2.0	1001.79	2.0	376.05

[a)] arbitrary units; cf. Supplemental Figure 3

3rd determination [a)]

ΔCV	RNAse S				S-Protein				S-Peptide			
[V]	1-19..21-124		1-20..22-124		21-124		22-124		1-19		1-20	
	$m_{(1)}+$	$h_{(1)}$	$m_{(2)}+$	$h_{(2)}$	$p_{(1)}+$	$j_{(1)}$	$p_{(2)}+$	$j_{(2)}$	$n_{(1)}+$	$i_{(1)}$	$n_{(2)}+$	$i_{(2)}$
**3**	6.7	252.93	6.7	137.83	5.4	22.03	5.1	46.63	2.0	4.85	2.0	1.10
**8**	6.7	335.64	6.7	185.63	5.5	24.39	5.1	61.49	2.0	11.19	2.0	3.34
**11**	6.6	442.58	6.6	241.10	5.2	102.71	5.1	89.05	2.0	67.49	2.0	17.90
**13**	6.6	262.10	6.5	141.46	5.1	177.11	5.1	133.90	2.0	142.17	2.0	37.82
**15**	6.5	191.03	6.4	112.23	5.1	269.79	5.1	185.77	2.0	240.13	2.0	69.57
**17**	6.4	204.35	6.3	125.36	5.0	381.48	5.0	254.70	2.0	386.03	2.0	125.91
**20**	6.3	205.45	6.2	125.59	5.0	447.04	5.0	301.45	2.0	511.48	2.0	170.84
**30**	6.2	116.56	6.2	63.27	4.9	528.84	4.9	342.64	2.0	839.67	2.0	292.85
**50**	6.3	40.40	6.3	17.97	4.9	589.00	4.9	358.39	2.0	977.17	2.0	351.83

[a)] arbitrary units; cf. Supplemental Figure 3

Supplemental Table 11: Ion intensities, charge states, and m/z values for RNAse S at various collision cell voltage difference settings in 40% methanol/200 mM ammonium acetate (pH 7).

1st determination

RNAse S Complex 1-19...21-124

ion	m/z	3V	8V	11V	13V	15V	17V	20V	30V	50V
5+	2727.74	4.23	5.26	6.33	7.17	18.10	16.40	15.00	10.60	4.12
6+	2272.74	128.00	183.00	175.00	192.00	88.40	119.00	167.00	96.70	40.80
7+	1948.30	349.00	449.00	389.00	263.00	208.00	197.00	148.00	60.40	30.00
8+	1704.76	2.46	2.40	1.57	0.84	5.60	4.74	1.06	0.76	0.00
9+	1515.49	0.00	0.00	0.29	0.00	0.00	0.00	0.00	0.00	0.00

RNAse S Complex 1-20...22-124

ion	m/z	3V	8V	11V	13V	15V	17V	20V	30V	50V
5+	2709.87	0.00	2.25	2.54	3.39	0.00	9.82	7.59	6.40	2.63
6+	2258.22	77.80	117.00	110.00	120.00	60.40	79.50	102.00	55.50	17.60
7+	1935.73	185.00	247.00	226.00	147.00	111.00	108.00	80.80	36.90	22.30
8+	1693.87	1.84	1.22	1.22	0.59	3.58	2.88	0.94	0.55	0.00
9+	1505.78	0.00	0.00	0.30	0.00	0.00	0.00	0.00	0.00	0.00

S Protein 21-124

ion	m/z	3V	8V	11V	13V	15V	17V	20V	30V	50V
3+	3845.75	0.00	0.00	0.00	0.00	0.00	0.00	0.00	0.00	0.00
4+	2884.80	0.00	0.00	0.00	0.00	0.00	0.00	0.00	5.77	65.00
5+	2307.98	22.20	24.60	86.10	196.00	130.00	252.00	459.00	498.00	637.00
6+	1923.31	32.90	45.20	46.60	39.10	33.20	42.40	44.50	42.50	54.40
7+	1648.86	1.43	1.36	1.69	1.67	3.16	3.72	2.53	2.47	1.43

S Protein 22-124

ion	m/z	3V	8V	11V	13V	15V	17V	20V	30V	50V
3+	3816.46	0.00	0.00	0.00	0.00	0.00	0.00	0.00	0.00	0.00
4+	2862.75	0.00	0.00	0.00	0.00	0.00	0.00	0.00	24.60	45.40
5+	2288.54	57.10	61.00	52.40	136.00	93.10	176.00	300.00	322.00	389.00
6+	1908.95	16.30	21.10	25.90	23.00	20.80	22.50	26.20	27.90	37.40
7+	1636.56	0.75	1.16	1.67	1.02	2.50	2.39	1.78	1.57	0.49

S Peptide 1-19

ion	m/z	3V	8V	11V	13V	15V	17V	20V	30V	50V
1+D	4189.30	0.00	0.00	0.00	0.00	0.00	0.00	0.00	0.00	0.00
1+	2095.33	0.00	0.00	0.00	0.00	0.00	0.00	0.00	5.46	13.40
2+	1048.15	12.70	27.60	71.10	151.00	202.00	342.00	612.00	921.00	1175.00
3+	699.13	0.50	0.84	0.56	0.27	3.58	4.26	1.48	1.90	2.30
4+	524.53	0.00	0.00	0.00	0.00	0.00	0.00	0.00	0.00	0.00

S Peptide 1-20

ion	m/z	3V	8V	11V	13V	15V	17V	20V	30V	50V
1+D	4330.36	0.00	0.00	0.00	0.00	0.00	0.00	0.00	0.00	0.00
1+	2166.40	0.00	0.00	0.00	0.00	0.00	0.00	0.00	0.00	5.03
2+	1083.70	2.90	6.77	17.40	45.90	62.60	106.00	196.00	330.00	436.00
3+	722.89	0.00	0.34	0.18	0.28	2.30	1.63	0.80	0.70	0.43
4+	542.30	0.00	0.00	0.00	0.00	0.00	0.00	0.00	0.00	0.00

D=Dimer

Supplemental Table 11: continued

2nd determination

RNAse S Complex 1-19...21-124										
ion	m/z	3V	8V	11V	13V	15V	17V	20V	30V	50V
5+	2727.82	4.81	5.65	9.89	14.50	12.40	13.50	13.50	13.10	4.56
6+	2272.93	88.00	23.60	155.00	282.00	208.00	231.00	237.00	138.00	48.50
7+	1948.39	282.00	231.00	609.00	552.00	284.00	208.00	140.00	65.60	34.10
8+	1705.01	2.26	4.38	2.28	2.06	0.86	0.65	0.71	0.00	0.00
9+	1515.49	0.00	0.00	0.00	0.00	0.00	0.00	0.00	0.00	0.00

RNAse S Complex 1-20...22-124										
ion	m/z	3V	8V	11V	13V	15V	17V	20V	30V	50V
5+	2709.72	1.17	3.23	4.32	6.47	5.27	6.87	9.09	7.19	2.74
6+	2258.43	55.70	44.30	97.40	175.00	132.00	148.00	149.00	75.90	18.50
7+	1935.97	158.00	127.00	349.00	298.00	149.00	116.00	75.90	39.50	20.60
8+	1693.89	1.07	2.49	1.28	1.16	0.62	0.61	0.63	0.00	0.38
9+	1505.76	0.00	0.00	0.00	0.00	0.00	0.00	0.00	0.00	0.00

S Protein 21-124										
ion	m/z	3V	8V	11V	13V	15V	17V	20V	30V	50V
3+	3845.75	0.00	0.00	0.00	0.00	0.00	0.00	0.00	0.00	0.00
4+	2884.80	0.00	0.00	0.00	0.00	0.00	0.00	4.94	62.30	95.10
5+	2308.36	17.50	22.70	89.30	319.00	327.00	470.00	601.00	688.00	725.00
6+	1923.67	25.10	21.70	53.80	59.50	39.80	45.30	46.20	46.20	54.30
7+	1648.96	0.87	1.80	2.79	1.98	1.47	1.59	2.18	1.95	0.90

S Protein 22-124										
ion	m/z	3V	8V	11V	13V	15V	17V	20V	30V	50V
3+	3816.46	0.00	0.00	0.00	0.00	0.00	0.00	0.00	0.00	0.00
4+	2862.98	0.00	0.00	0.00	0.00	0.00	0.00	4.84	43.50	63.00
5+	2288.79	43.00	35.60	65.30	226.00	231.00	327.00	389.00	421.00	435.00
6+	1908.90	9.88	11.50	23.50	30.30	22.10	25.40	3.41	28.60	31.90
7+	1636.41	0.61	1.14	1.40	1.50	0.96	0.86	1.16	0.94	0.77

S Peptide 1-19										
ion	m/z	3V	8V	11V	13V	15V	17V	20V	30V	50V
1+D	4189.30	0.00	0.00	0.00	0.00	0.00	0.00	0.00	0.00	0.00
1+	2095.33	0.00	0.00	0.00	0.00	0.00	0.00	0.00	8.46	16.20
2+	1048.26	13.70	68.10	114.00	289.00	297.00	456.00	644.00	1011.00	1180.00
3+	699.27	0.06	1.37	0.90	1.27	1.31	0.85	1.31	1.54	1.61
4+	524.53	0.00	0.00	0.00	0.00	0.00	0.00	0.00	0.00	0.00

S Peptide 1-20										
ion	m/z	3V	8V	11V	13V	15V	17V	20V	30V	50V
1+D	4330.36	0.00	0.00	0.00	0.00	0.00	0.00	0.00	0.00	0.00
1+	2166.25	0.00	0.00	0.00	0.00	0.00	0.00	0.00	6.10	6.81
2+	1083.75	3.52	20.10	27.90	77.20	83.00	154.00	222.00	368.00	440.00
3+	722.95	0.00	1.06	0.23	0.70	0.29	0.35	0.56	0.28	0.61
4+	542.30	0.00	0.00	0.00	0.00	0.00	0.00	0.00	0.00	0.00

D=Dimer

Supplemental Table 11: continued

3rd determination

RNAse S Complex 1-19...21-124										
ion	m/z	3V	8V	11V	13V	15V	17V	20V	30V	50V
5+	2727.45	4.28	6.07	9.20	11.50	12.80	13.10	13.60	13.80	4.56
6+	2272.83	113.00	161.00	190.00	199.00	207.00	218.00	219.00	140.00	51.50
7+	1948.30	291.00	424.00	415.00	327.00	238.00	171.00	118.00	61.20	31.20
8+	1705.18	2.06	1.79	1.68	1.35	1.16	0.00	0.00	0.00	0.00
9+	1515.49	0.00	0.00	0.00	0.00	0.00	0.00	0.00	0.00	0.00

RNAse S Complex 1-20...22-124										
ion	m/z	3V	8V	11V	13V	15V	17V	20V	30V	50V
5+	2709.52	1.51	2.17	3.03	3.91	5.56	5.88	7.29	8.15	3.19
6+	2258.25	67.70	101.00	120.00	124.00	133.00	136.00	133.00	75.40	19.10
7+	1935.81	165.00	233.00	235.00	181.00	129.00	89.40	63.10	37.40	21.20
8+	1694.07	1.23	0.99	1.48	1.04	0.67	0.00	0.00	0.00	0.00
9+	1505.76	0.00	0.00	0.00	0.00	0.00	0.00	0.00	0.00	0.00

S Protein 21-124										
ion	m/z	3V	8V	11V	13V	15V	17V	20V	30V	50V
3+	3845.75	0.00	0.00	0.00	0.00	0.00	0.00	0.00	0.00	0.00
4+	2884.78	0.00	0.00	0.00	0.00	0.00	0.00	4.45	72.10	108.00
5+	2308.15	18.00	27.40	89.50	204.00	336.00	425.00	522.00	679.00	741.00
6+	1923.57	29.00	41.20	44.80	48.70	42.10	38.80	37.60	36.80	45.50
7+	1648.95	1.15	1.27	1.78	1.73	1.59	1.20	1.15	1.42	0.00

S Protein 22-124										
ion	m/z	3V	8V	11V	13V	15V	17V	20V	30V	50V
3+	3816.46	0.00	0.00	0.00	0.00	0.00	0.00	0.00	0.00	0.00
4+	2862.98	0.00	0.00	0.00	0.00	0.00	0.00	4.10	48.50	69.70
5+	2290.68	50.10	66.20	72.90	75.70	233.00	289.00	346.00	427.00	453.00
6+	1909.10	13.00	16.60	22.50	25.10	21.70	20.40	22.70	23.10	30.70
7+	1636.31	0.53	1.01	1.01	0.93	0.94	0.19	0.57	0.55	0.00

S Peptide 1-19										
ion	m/z	3V	8V	11V	13V	15V	17V	20V	30V	50V
1+D	4189.30	0.00	0.00	0.00	0.00	0.00	0.00	0.00	0.00	0.00
1+	2095.35	0.00	0.00	0.00	0.00	0.00	0.00	0.00	7.08	15.70
2+	1048.18	9.06	21.60	70.00	179.00	299.00	440.00	570.00	985.00	1166.00
3+	699.16	0.14	0.42	0.39	0.57	0.48	0.84	0.79	2.22	2.14
4+	524.53	0.00	0.00	0.00	0.00	0.00	0.00	0.00	0.00	0.00

S Peptide 1-20										
ion	m/z	3V	8V	11V	13V	15V	17V	20V	30V	50V
1+D	4330.36	0.00	0.00	0.00	0.00	0.00	0.00	0.00	0.00	0.00
1+	2166.39	0.00	0.00	0.00	0.00	0.00	0.00	0.00	0.00	6.22
2+	1083.70	2.37	6.08	18.60	48.70	88.80	144.00	195.00	343.00	433.00
3+	722.88	0.31	0.00	0.23	0.30	0.07	0.28	0.51	0.87	0.85
4+	542.30	0.00	0.00	0.00	0.00	0.00	0.00	0.00	0.00	0.00

D=Dimer

Supplemental Table 12: Apex heights and mean charge states of educt and product ion signals upon gas phase dissociation of RNAse S dissolved in 40 % MeOH / 200 mM ammonium acetate (pH 7).

1st determination [a)]

ΔCV	RNAse S				S-Protein				S-Peptide			
[V]	1-19..21-124		1-20..22-124		21-124		22-124		1-19		1-20	
	$m_{(1)}+$	$h_{(1)}$	$m_{(2)}+$	$h_{(2)}$	$p_{(1)}+$	$j_{(1)}$	$p_{(2)}+$	$j_{(2)}$	$n_{(1)}+$	$i_{(1)}$	$n_{(2)}+$	$i_{(2)}$
**3**	6.7	348.34	6.7	187.64	5.6	33.74	5.2	57.50	2.0	12.94	2.0	2.96
**8**	6.7	449.35	6.7	250.49	5.7	45.90	5.3	61.58	2.0	28.19	2.0	6.87
**11**	6.7	390.95	6.7	226.50	5.4	88.27	5.4	53.76	2.0	73.13	2.0	17.49
**13**	6.6	268.67	6.5	152.87	5.2	196.77	5.2	136.35	2.0	151.75	2.0	46.90
**15**	6.6	210.94	6.7	111.35	5.2	131.09	5.2	93.83	2.0	207.13	2.0	63.81
**17**	6.6	203.80	6.5	110.96	5.2	253.05	5.1	176.50	2.0	341.95	2.0	108.54
**20**	6.4	169.71	6.4	103.90	5.1	459.10	5.1	300.09	2.0	620.75	2.0	200.16
**30**	6.3	99.08	6.3	56.38	5.1	504.87	5.0	328.46	2.0	929.62	2.0	336.85
**50**	6.4	41.94	6.5	22.92	5.0	641.98	5.0	397.54	2.0	1172.99	2.0	440.97

[a)] arbitrary units; cf. Supplemental Figure 4

2nd determination [a)]

ΔCV	RNAse S				S-Protein				S-Peptide			
[V]	1-19..21-124		1-20..22-124		21-124		22-124		1-19		1-20	
	$m_{(1)}+$	$h_{(1)}$	$m_{(2)}+$	$h_{(2)}$	$p_{(1)}+$	$j_{(1)}$	$p_{(2)}+$	$j_{(2)}$	$n_{(1)}+$	$i_{(1)}$	$n_{(2)}+$	$i_{(2)}$
**3**	6.8	281.60	6.7	157.30	5.6	25.43	5.2	43.25	2.0	13.90	2.0	3.53
**8**	6.9	230.18	6.7	129.04	5.6	22.91	5.3	35.99	2.0	68.73	2.0	20.38
**11**	6.8	607.50	6.8	351.30	5.4	91.00	5.3	65.96	2.0	114.82	2.0	28.51
**13**	6.6	553.23	6.6	302.66	5.2	319.97	5.1	226.31	2.0	290.53	2.0	78.24
**15**	6.5	290.73	6.5	151.32	5.1	327.19	5.1	230.98	2.0	298.58	2.0	83.98
**17**	6.4	230.93	6.4	150.80	5.1	469.84	5.1	326.76	2.0	458.12	2.0	154.56
**20**	6.3	240.23	6.3	150.41	5.1	606.91	5.0	395.31	2.0	647.16	2.0	221.67
**30**	6.2	138.91	6.3	76.66	5.0	694.52	5.0	429.41	2.0	1012.94	2.0	370.28
**50**	6.3	49.24	6.4	20.99	5.0	731.60	4.9	435.85	2.0	1186.95	2.0	442.40

[a)] arbitrary units; cf. Supplemental Figure 4

3rd determination [a)]

ΔCV	RNAse S				S-Protein				S-Peptide			
[V]	1-19..21-124		1-20..22-124		21-124		22-124		1-19		1-20	
	$m_{(1)}+$	$h_{(1)}$	$m_{(2)}+$	$h_{(2)}$	$p_{(1)}+$	$j_{(1)}$	$p_{(2)}+$	$j_{(2)}$	$n_{(1)}+$	$i_{(1)}$	$n_{(2)}+$	$i_{(2)}$
**3**	6.7	291.86	6.7	164.60	5.7	29.14	5.2	50.41	2.0	9.03	2.1	2.34
**8**	6.7	422.41	6.7	235.46	5.6	41.38	5.2	66.65	2.0	21.48	2.0	6.14
**11**	6.7	417.83	6.7	236.57	5.4	90.27	5.3	73.49	2.0	70.40	2.0	18.62
**13**	6.6	326.84	6.6	180.62	5.2	205.80	5.3	76.34	2.0	178.84	2.0	48.85
**15**	6.5	241.58	6.5	133.55	5.1	336.25	5.1	232.94	2.0	298.27	2.0	88.65
**17**	6.4	218.19	6.4	136.03	5.1	424.75	5.1	288.57	2.0	439.20	2.0	143.88
**20**	6.3	218.42	6.3	131.72	5.1	527.16	5.1	350.71	2.0	569.92	2.0	194.27
**30**	6.2	141.38	6.2	75.26	5.0	676.47	5.0	424.41	2.0	979.86	2.0	340.08
**50**	6.3	51.41	6.4	21.31	4.9	735.31	4.9	446.82	2.0	1167.64	2.0	432.96

[a)] arbitrary units; cf. Supplemental Figure 4

Supplemental Table 13: Distances of atoms of S-peptide amino acid residues to contact residues on the S-protein of RNase S in bulk solution simulations [a,b,c)].

S-peptide		S-protein		distance after simulation time [Å]			atom pair	S-peptide residue		
residue	atom	residue	atom	0 ns [d)]	100 ns	200 ns	contact counts	contact positions	sum of contacts	position to sum ratio
A4	CB	V116	CG1		3.687		1	5	5	1,00
A4	CB	V116	CG2			3.623	1			
A4	CB	P117	O		3.897		1			
A4	CB	V118	CB	3.995			1			
A4	CB	V118	CG1	3.810			1			
A5	CA	P117	O	3.685			1	4	4	1,00
A5	CB	P117	O	3.944			1			
A5	CB	V116	CG2	3.616			1			
A5	CB	V116	CG1		3.648		1			
F8	CB	P117	O		3.660		1	11	15	1,36
F8	CD1	H119	CA	3.930			1			
F8	CD2	P117	O		3.822	3.301	2			
F8	CD2	P117	C		3.863	3.836	2			
F8	CD2	P117	CB		3.842		1			
F8	CE1	V54	CG1			3.756	1			
F8	CE1	P117	CB	3.962			1			
F8	CE2	P117	CB	3.803	3.781	3.965	3			
F8	CG	P117	O		3.922		1			
F8	CZ	V108	CG1			3.868	1			
F8	CZ	P117	CB	3.739			1			
E9	CG	L51	CD1	3.708			1	8	8	1,00
E9	CD	R33	NH2			3.664	1			
E9	CD	L51	CD2	3.800			1			
E9	OE1	R33	NH2			3.477	1			
E9	OE1	L51	CG	3.950			1			
E9	OE1	L51	CD1	3.633			1			
E9	OE1	L51	CD2	3.264			1			
E9	OE1	Q55	NE2	3.706			1			
R10	CA	R33	NH2	3.738			1	16	16	1,00
R10	CG	R33	O	3.792			1			
R10	CD	R33	O	3.733			1			
R10	CZ	R33	O	3.942			1			
R10	CZ	R33	CG			3.943	1			
R10	NH1	S32	O			3.980	1			
R10	NH1	R33	CG			3.860	1			
R10	NH2	R33	C	3.995			1			
R10	NH2	R33	O	2.805			1			
R10	NH2	R33	CZ			3.470	1			
R10	NH2	R33	NH1			3.394	1			
R10	NH2	R33	NH2			3.697	1			
R10	NH2	N34	CB	3.712			1			
R10	NH2	N34	CG	3.852			1			
R10	NH2	N34	OD1	3.404			1			
R10	NH2	N34	O	3.855			1			
Q11	CB	N44	ND2		3.885		1	4	4	1,00
Q11	CG	L35	CD2		3.645		1			
Q11	CG	L35	CD	3.696			1			
Q11	NE2	K41	CE	3.512			1			
H12	CB	T45	O	3.629			1	25	32	1,28
H12	CG	T45	O	3.669			1			
H12	CG	H119	CG		3.967		1			
H12	CG	F120	CB		3.956	3.364	2			
H12	CD2	V118	O		3.978		1			
H12	CD2	H119	CA		3.905	3.666	2			
H12	CD2	H119	C			3.761	1			
H12	CD2	F120	N		3.874	3.417	2			
H12	CD2	F120	CB			3.828	1			
H12	CE1	T45	O	3.956			1			
H12	CE1	N44	ND2		3.773		1			
H12	CE1	F120	CB	3.860		3.737	2			
H12	CE1	F120	N		3.663	3.577	2			
H12	CE1	F120	O			3.570	1			
H12	ND1	N44	ND2		3.963		1			
H12	ND1	T45	O	2.891			1			
H12	ND1	F120	N		3.943		1			

S-peptide		S-protein		distance after simulation time [Å]			atom pair	S-peptide residue		
residue	atom	residue	atom	0 ns [d)]	100 ns	200 ns	contact counts	contact positions	sum of contacts	position to sum ratio
H12	ND1	F120	CB		3.720		1			
H12	ND1	F120	CD1			3.914	1			
H12	NE2	V118	O		3.915		1			
H12	NE2	H119	CA		3.603	3.424	2			
H12	NE2	H119	C		3.995		1			
H12	NE2	H119	ND1		3.987		1			
H12	NE2	F120	N		3.619	3.253	2			
H12	NE2	F120	CB	3.784			1			
M13	CB	V47	O			3.497	1	8	10	1,25
M13	CE	V47	O	3.776			1			
M13	CE	E49	O	3.092			1			
M13	CE	L51	N	3.963			1			
M13	CE	L51	CA	3.950			1			
M13	CG	V47	O		3.587	3.272	2			
M13	CG	L51	CD1	3.981			1			
M13	SD	L51	CD1		3.805	3.844	2			
D14	CA	V47	O	3.621			1	11	16	1,45
D14	CB	Y25	OH	3.656			1			
D14	CB	M29	CE			3.651	1			
D14	CB	R33	NH2		3.679	3.667	2			
D14	CB	F46	CB		3.861		1			
D14	CG	M29	CE	3.656	3.494		2			
D14	CG	R33	NH2		3.523	3.599	2			
D14	OD1	M29	CE	3.475			1			
D14	OD1	R33	CZ			3.640	1			
D14	OD1	R33	NH1		3.346	3.717	2			
D14	OD1	R33	NH2		2.572	2.769	2			
S15	CA	E49	O	3.546			1	2	2	1,00
S15	CB	S50	CA	3.991			1			

a) contacts between paired atoms are assumed when distances are < 4 Å
b) atoms with distances > 4 Å to next neighbor atoms are not listed.
c) atoms from amino S-peptide acid residues 16-20 are not considered
d) atom coordinates from 1KF5.pdb

Supplemental Table 14: Distances of atoms of S-peptide amino acid residues to contact residues on the S-protein of RNase S in evaporating droplet simulations [a,b,c].

S-peptide		S-protein		distance after simulation [Å]										atom pair	S-peptide residue		
residue	atom	residue	atom	model 01	model 02	model 03	model 04	model 05	model 06	model 07	model 08	model 09	model 10	contact counts	contact positions	sum of contacts	position-to-sum ratio
K1	C	D38	OD1	3.515										1	72	72	1,00
K1	N	G112	O		2.975									1			
K1	N	G112	C		3.456									1			
K1	N	G112	CA		3.842									1			
K1	CA	D38	OD1	3.363										1			
K1	CB	L51	CD2								3.939			1			
K1	CB	N71	CG							3.746				1			
K1	CB	N71	ND2							3.604				1			
K1	CB	N113	OD1		3.267									1			
K1	CD	D38	O	3.597										1			
K1	CD	D38	CA	3.844										1			
K1	CD	D38	OD1	3.421										1			
K1	CD	L51	CD2								3.990			1			
K1	CD	N71	CB							3.923				1			
K1	CD	P114	O				3.326							1			
K1	CD	T115	CE1							3.959				1			
K1	CD	H119	NE2		3.643									1			
K1	CE	D38	O	3.677										1			
K1	CE	S59	OG				3.230							1			
K1	CE	K61	NZ							3.542				1			
K1	CE	T70	CG2			3.883								1			
K1	CE	T70	N			3.895								1			
K1	CE	T70	OG1			3.430								1			
K1	CE	Y92	OH	3.789										1			
K1	CE	Q69	OE1		3.501									1			
K1	CE	P114	OH									3.854		1			
K1	CE	T115	O				3.743							1			
K1	CE	H119	NE2		3.834									1			
K1	CG	L51	CD2								3.755			1			
K1	CG	Q69	CD			3.981								1			
K1	NZ	K37	C	3.931										1			
K1	NZ	K37	O	2.854										1			
K1	NZ	D38	C	3.654										1			
K1	NZ	D38	CA	3.893										1			
K1	NZ	D38	O	2.727										1			
K1	NZ	H48	NE2										3.969	1			
K1	NZ	S50	OG						2.691					1			
K1	NZ	S50	CB						3.639					1			
K1	NZ	Q55	CD								3.626			1			
K1	NZ	C58	C				3.645							1			

S-peptide		S-protein		distance after simulation [Å]										atom pair	S-peptide residue		
residue	atom	residue	atom	model 01	model 02	model 03	model 04	model 05	model 06	model 07	model 08	model 09	model 10	contact counts	contact positions	sum of contacts	position-to-sum ratio
K1	NZ	C58	O				2.950							1			
K1	NZ	S59	CB				3.589							1			
K1	NZ	S59	OG				2.998							1			
K1	NZ	K61	CD							3.752				1			
K1	NZ	K61	CE							3.512				1			
K1	NZ	N62	CG							3.866				1			
K1	NZ	G68	O		2.657									1			
K1	NZ	G68	C		3.665									1			
K1	NZ	T70	OG1			2.828								1			
K1	NZ	T70	N			3.839								1			
K1	NZ	T73	CZ							3.621				1			
K1	NZ	T73	OH							2.956				1			
K1	NZ	T73	CE1							3.934				1			
K1	NZ	Y92	CE1	3.315										1			
K1	NZ	Y92	CZ	3.235										1			
K1	NZ	Y92	OH	2.663										1			
K1	NZ	Q69	CD		3.674									1			
K1	NZ	Q69	OE1		2.630									1			
K1	NZ	T70	CB			3.974								1			
K1	NZ	N71	CG			3.635								1			
K1	NZ	N71	ND2			3.939								1			
K1	NZ	G112	C									3.728		1			
K1	NZ	N113	CB									3.840		1			
K1	NZ	N113	CG									3.491		1			
K1	NZ	P114	O				3.021	3.263						1			
K1	NZ	T115	C				3.827	3.685						1			
K1	NZ	T115	CZ									3.501		1			
K1	NZ	T115	CE1									3.677		1			
K1	NZ	T115	OH									2.889		1			
K1	NZ	H119	CD2		3.394									1			
K1	NZ	H119	NE2		2.885									1			
K1	NZ	H119	CE1		3.930									1			
E2	CA	D38	OD1	3.998										1	47	47	1,00
E2	CB	R33	NH2								3.909			1			
E2	CB	R39	OD1	3.852										1			
E2	CB	N113	ND2		3.789									1			
E2	CB	N113	CG		3.605									1			
E2	CB	V118	CG2									3.619		1			
E2	CD	D38	HD2	3.028										1			
E2	CD	D38	OD2	3.963										1			
E2	CD	R39	CZ	3.625										1			
E2	CD	R39	NE	3.931										1			

S-peptide		S-protein		distance after simulation [Å]										atom pair	S-peptide residue		
residue	atom	residue	atom	model 01	model 02	model 03	model 04	model 05	model 06	model 07	model 08	model 09	model 10	contact counts	contact positions	sum of contacts	position-to-sum ratio
E2	CD	K66	NZ									3.812		1			
E2	CD	N67	ND2									3.573		1			
E2	CD	G68	O							3.547				1			
E2	CG	A109	CB									3.771		1			
E2	CG	H119	CB									3.986		1			
E2	OE1	D38	CG	3.657										1			
E2	OE1	D38	OD1	3.805										1			
E2	OE1	D38	OD2	2.822										1			
E2	OE1	R39	CZ	3.257										1			
E2	OE1	R39	NH1	3.516										1			
E2	OE1	R39	NH2	3.570										1			
E2	OE1	R39	NE	3.495										1			
E2	OE1	R39	CD	3.925										1			
E2	OE1	Q55	O							3.525				1			
E2	OE1	G68	CB				3.987							1			
E2	OE1	V116	CG2		3.785									1			
E2	OE1	V118	CG1		3.679									1			
E2	OE2	R39	NH1	2.713										1			
E2	OE2	R39	CZ	3.290										1			
E2	OE2	R39	NE	3.858										1			
E2	OE2	R39	CD	3.895										1			
E2	OE2	L51	CG				3.798							1			
E2	OE2	L51	CD1				3.375							1			
E2	OE2	N55	CG				3.914							1			
E2	OE2	K66	CD									3.676		1			
E2	OE2	K66	CE									3.740		1			
E2	OE2	K66	NZ									2.795		1			
E2	OE2	G68	C							3.904				1			
E2	OE2	G68	O							2.819				1			
E2	OE2	T70	OG1							3.629				1			
E2	OE2	A109	CB									3.848		1			
E2	OE2	P114	CD		3.478									1			
E2	OE2	P114	N		3.478									1			
E2	OE2	V116	CG2		3.806									1			
E2	OE2	H119	CB									3.239		1			
E2	OE2	H119	CG									3.482		1			
E2	OE2	H119	CD2									3.642		1			
T3	CB	V118	CG2							3.736				1	11	11	1,00
T3	CG2	L51	CD2										3.956	1			
T3	CG2	E111	CG2							3.815				1			
T3	CG2	V118	CG2							3.526				1			
T3	OG1	Q55	CG										3.988	1			

S-peptide		S-protein		distance after simulation [Å]										atom pair	S-peptide residue		
residue	atom	residue	atom	model 01	model 02	model 03	model 04	model 05	model 06	model 07	model 08	model 09	model 10	contact counts	contact positions	sum of contacts	position-to-sum ratio
T3	OG1	N113	C				3.546							1			
T3	OG1	P114	N				3.866							1			
T3	OG1	P114	CD				3.655							1			
T3	OG1	V116	CG1					3.462						1			
T3	OG1	V116	CG2				3.571							1			
T3	OG1	V118	CG2							3.929				1			
A4	CB	Q55	OE1										3.641	1	6	6	1,00
A4	CB	Q69	CG							3.881				1			
A4	CB	P114	CG										3.656	1			
A4	CB	V118	O		3.619									1			
A4	CB	H119	CE1							3.563				1			
A4	CB	H119	NE2							3.496				1			
A5	CA	V118	CG1								3.815			1	8	8	1,00
A5	CB	Q55	CD										3.638	1			
A5	CB	V116	CB					3.842					3.842	1			
A5	CB	V116	CG1					3.722					3.465	1			
A5	CB	V116	CG2								3.983			1			
A5	CB	P117	CD										3.903	1			
A5	CB	P117	O								3.602			1			
A5	CB	V118	CG1								3.815			1			
A6	CB	L51	CD1						3.907					1	5	5	1,00
A6	CB	Q55	OE1						3.724					1			
A6	CB	V116	CB						3.987					1			
A6	CA	N113	O			3.306								1			
A6	CA	V118	CG1									3.954		1			
K7	CB	V118	CB						3.965					1	34	46	1,35
K7	CB	V118	CG1						3.477					1			
K7	CD	K37	NZ									3.598		1			
K7	CE	R33	O			3.885								1			
K7	CE	N34	O		3.531									1			
K7	CE	K37	NZ									3.371		1			
K7	CE	R39	NH1		3.710	3.396								1			
K7	CE	R39	NH2		3.540									1			
K7	CE	Q69	OE1	3.712										1			
K7	CE	H119	CD2	3.680										1			
K7	CE	H119	NE2	3.502				3.727					3.564	1			
K7	CG	H119	CD2	3.865										1			
K7	NZ	R33	O			2.751						3.766		2			
K7	NZ	R33	C			3.940								1			
K7	NZ	N34	O		3.832									1			
K7	NZ	N34	CG									3.779		1			
K7	NZ	K37	O		2.997									1			

S-peptide		S-protein		distance after simulation [Å]										atom pair	S-peptide residue		
residue	atom	residue	atom	model 01	model 02	model 03	model 04	model 05	model 06	model 07	model 08	model 09	model 10	contact counts	contact positions	sum of contacts	position-to-sum ratio
K7	NZ	K37	NZ									3.167		1			
K7	NZ	K37	CE									3.867		1			
K7	NZ	R39	NH1		2.747	2.824								2			
K7	NZ	R39	NH2		3.104									1			
K7	NZ	R39	CZ		3.272	3.846								2			
K7	NZ	G68	C								3.767			1			
K7	NZ	Q69	CD								3.716			1			
K7	NZ	Q69	OE1	2.630										1			
K7	NZ	Q69	CD	3.576				3.854						2			
K7	NZ	Q69	CG	3.911										1			
K7	NZ	V118	O	3.356										1			
K7	NZ	V118	C					3.809					3.829	2			
K7	NZ	H119	NE2	3.232				2.930					2.825	3			
K7	NZ	H119	CE1	3.581				3.533						2			
K7	NZ	H119	ND1	3.575				3.838						2			
K7	NZ	H119	CG	3.400				3.593						2			
K7	NZ	H119	CD2	3.179				3.047					3.080	3			
F8	CB	N55	NE2				3.923							1	25	29	1,16
F8	CB	P117	O		3.976									1			
F8	CB	P117	CB						3.770					1			
F8	CD1	V116	CG2			3.924								1			
F8	CD1	P117	O		3.586									1			
F8	CD1	P117	CB								3.836			1			
F8	CD1	P117	CG										3.756	1			
F8	CD2	V108	CG1							3.990				1			
F8	CD2	P117	C				3.963							1			
F8	CD2	F120	CB							3.985				1			
F8	CE1	V47	CG2						3.486			3.745		2			
F8	CE1	V54	CG1				3.618					3.775		2			
F8	CE1	V108	CG1					3.424						1			
F8	CE1	P117	CB		3.984					3.866				2			
F8	CE1	P117	CE1										3.888	1			
F8	CE1	P117	O		3.987									1			
F8	CE1	F120	CB					3.964						1			
F8	CE1	F120	HD1					3.222						1			
F8	CE2	F120	CB		3.870						3.929			2			
F8	CE2	F120	CG2								3.601			1			
F8	CG	Q55	NE2				3.843							1			
F8	CG	V116	CG2			3.757								1			
F8	CG	P117	O		3.842									1			
F8	CZ	V108	CG1					3.812						1			
F8	CZ	F120	CB		3.732									1			

S-peptide residue	S-peptide atom	S-protein residue	S-protein atom	distance after simulation [Å] model 01	model 02	model 03	model 04	model 05	model 06	model 07	model 08	model 09	model 10	atom pair contact counts	S-peptide residue contact positions	S-peptide residue sum of contacts	S-peptide residue position-to-sum ratio
E9	CD	R33	NH1				3.681							1	20	21	1,05
E9	CD	Q55	OE1	3.610										1			
E9	CD	P114	CD			3.820								1			
E9	OE1	R33	NH1				2.938							1			
E9	OE1	L51	N						3.364					1			
E9	OE1	L51	CB						3.503					1			
E9	OE1	V54	CG2								3.919			1			
E9	OE1	Q55	OE1	3.697										1			
E9	OE1	P114	CD			3.333								1			
E9	OE1	P117	CD					3.953						1			
E9	OE1	P117	CG					3.505						1			
E9	OE1	V118	CA							3.739				1			
E9	OE1	V118	O							3.801				1			
E9	OE2	V54	O								3.086			1			
E9	OE2	Q55	OE1	2.674							3.716			2			
E9	OE2	Q55	NE2	3.576										1			
E9	OE2	Q55	CD	3.522										1			
E9	OE2	V116	CG1							3.776				1			
E9	OE2	P117	C							3.815				1			
E9	OE2	P117	CG								3.776			1			
R10	C	R33	NH2										3.952	1	47	53	1,13
R10	O	R33	NH1										3.001	1			
R10	O	R33	NH2										2.831	1			
R10	CB	R33	CB							3.662				1			
R10	CB	R33	CG							3.741				1			
R10	CD	R33	CD								3.840			1			
R10	CD	R33	NH1					3.793						1			
R10	CD	R33	NH2			3.990								1			
R10	CG	L35	CD2	3.759										1			
R10	CZ	R33	CA		3.495									1			
R10	CZ	R33	CD									3.949		1			
R10	CZ	R33	CZ		3.904							3.915		2			
R10	CZ	R33	NE		3.916	3.908								2			
R10	CZ	R33	CG			3.427						3.944		2			
R10	CZ	R33	NH1					3.960				3.717		1			
R10	CZ	L35	CD2							3.991				1			
R10	NE	R33	CG			3.629								1			
R10	NE	R33	O							3.868				1			
R10	NH1	S32	O		3.031									1			
R10	NH1	R33	CA		3.420									1			
R10	NH1	R33	C		3.468									1			
R10	NH1	R33	CZ			3.648		3.852						2			

S-peptide		S-protein		distance after simulation [Å]										atom pair	S-peptide residue		
residue	atom	residue	atom	model 01	model 02	model 03	model 04	model 05	model 06	model 07	model 08	model 09	model 10	contact counts	contact positions	sum of contacts	position-to-sum ratio
R10	NH1	R33	NE			3.160								1			
R10	NH1	R33	NH1			3.566								1			
R10	NH1	R33	NH2			3.071								1			
R10	NH1	R33	CD			3.933								1			
R10	NH1	N34	CG		3.610									1			
R10	NH1	L35	CD2				2.993							1			
R10	NH1	D38	CG				3.963							1			
R10	NH1	D38	OD2				3.210							1			
R10	NH2	S32	C		3.943			3.776						2			
R10	NH2	S32	O					2.795						1			
R10	NH2	S32	CB			3.771								1			
R10	NH2	S32	OG			3.406								1			
R10	NH2	R33	CA		3.839			3.503						2			
R10	NH2	R33	CD									3.993		1			
R10	NH2	R33	CG			3.703								1			
R10	NH2	R33	CZ									3.119		1			
R10	NH2	R33	O					3.934						1			
R10	NH2	R33	C					3.943						1			
R10	NH2	R33	NH1									2.983		1			
R10	NH2	R33	NH2									3.698		1			
R10	NH2	R33	NE									3.605		1			
R10	NH2	N34	OD1					3.233						1			
R10	NH2	D38	CG	3.922										1			
R10	NH2	D38	OD2	3.995						3.595				1			
R10	NH2	L35	CD2							3.137				1			
Q11	CB	L35	CD2		3.972									1	35	43	1,23
Q11	CD	L35	CD2		3.853	3.790	3.945							3			
Q11	CD	K37	NZ									3.650		1			
Q11	CD	D38	OD2	3.141							3.521			2			
Q11	CD	D38	CB	3.698										1			
Q11	CD	D38	CG	3.900							3.777			2			
Q11	CD	R39	NH2			3.694								1			
Q11	CG	D38	CB									3.921		1			
Q11	CG	D38	CG									3.763		1			
Q11	CG	D38	OD2	3.702								3.236		2			
Q11	NE2	L35	CB	3.681										1			
Q11	NE2	L35	CD1		3.754									1			
Q11	NE2	L35	CD2			3.180	3.992							2			
Q11	NE2	L35	CG		3.974									1			
Q11	NE2	L35	CB		3.688									1			
Q11	NE2	K37	NZ									3.561		1			
Q11	NE2	D38	CA									3.947		1			

S-peptide		S-protein		distance after simulation [Å]										atom pair	S-peptide residue		
residue	atom	residue	atom	model 01	model 02	model 03	model 04	model 05	model 06	model 07	model 08	model 09	model 10	contact counts	contact positions	sum of contacts	position-to-sum ratio
Q11	NE2	D38	CB									3.880		1			
Q11	NE2	D38	CG								3.529			1			
Q11	NE2	D38	N									3.657		1			
Q11	NE2	D38	OD2								3.744			1			
Q11	NE2	R39	CZ			3.931								1			
Q11	NE2	R39	NH2	3.985		3.527								2			
Q11	NE2	K41	CB		3.858									1			
Q11	NE2	K41	CD		3.799									1			
Q11	NE2	N44	CG				3.419			3.531				2			
Q11	NE2	N44	ND2				3.648							1			
Q11	NE2	N44	OD1				2.704							1			
Q11	NE2	V118	O						2.834					1			
Q11	NE2	V118	C						3.960					1			
Q11	OE1	L35	CD2				3.596							1			
Q11	OE1	D38	OD2	3.294										1			
Q11	OE1	D38	CB	3.067										1			
Q11	OE1	D38	CG	3.605										1			
Q11	OE1	R39	NH2			3.267								1			
H12	CB	F120	CB				3.872			3.777				2	72	87	1,21
H12	CD2	L35	CD1						3.742					1			
H12	CD2	R38	CB									3.700		1			
H12	CD2	R38	CG									3.768		1			
H12	CD2	R39	CZ								3.541		3.988	2			
H12	CD2	R39	NH1								3.658			1			
H12	CD2	R39	NH2								3.283		3.795	2			
H12	CD2	N44	CA			3.913								1			
H12	CD2	N44	C			3.998								1			
H12	CD2	T45	O					3.848						1			
H12	CD2	F46	CE1									3.528		1			
H12	CD2	F120	CB					3.718					3.611	2			
H12	CD2	F120	CG					3.722						1			
H12	CD2	F120	CD1					3.848		3.861	3.746			3			
H12	CD2	F120	CE1								3.553			1			
H12	CE1	R39	CZ										3.907	1			
H12	CE1	K41	CD	3.645	3.790									2			
H12	CE1	K41	CE	3.787	3.986									2			
H12	CE1	K41	NZ	3.800	3.820									2			
H12	CE1	N44	CG		3.742									1			
H12	CE1	T45	O			3.098								1			
H12	CE1	T45	OG1								3.840			1			
H12	CE1	H119	CA				3.705						3.833	2			
H12	CE1	H119	CG									3.975		1			

S-peptide		S-protein		distance after simulation [Å]										atom pair	S-peptide residue		
residue	atom	residue	atom	model 01	model 02	model 03	model 04	model 05	model 06	model 07	model 08	model 09	model 10	contact counts	contact positions	sum of contacts	position-to-sum ratio
H12	CE1	H119	CD2						3.953					1			
H12	CE1	H119	ND1									3.932		1			
H12	CE1	H119	CE1									3.603		1			
H12	CE1	H119	NE2								3.763			1			
H12	CE1	F120	N										3.490	1			
H12	CE1	F120	O								3.063			1			
H12	CE1	F120	CG		3.576									1			
H12	CE1	F120	CD1		3.208									1			
H12	CE1	F120	CE1		3.607									1			
H12	CG	R39	NH1								3.737			1			
H12	CG	T45	O			3.833								1			
H12	CG	F46	CE1									3.935		1			
H12	CG	F120	CB	3.636						3.572				2			
H12	CG	F120	CD1								3.874			1			
H12	CG	F120	CE1						3.621					1			
H12	ND1	K41	CD	3.652										1			
H12	ND1	K41	NZ	3.075										1			
H12	ND1	K41	CE	3.548										1			
H12	ND1	N44	CG		3.867									1			
H12	ND1	T45	O			3.182								1			
H12	ND1	H119	CE1								3.706			1			
H12	ND1	H119	CD2								3.573			1			
H12	ND1	H119	NE2								3.002			1			
H12	ND1	F120	CB	3.962						3.676				1			
H12	ND1	F120	O	3.765							2.932			1			
H12	ND1	F120	CD1		3.993									1			
H12	ND1	F120	CE1						3.768					1			
H12	NE2	D38	OD2									3.880		1			
H12	NE2	R39	CD										3.664	1			
H12	NE2	R39	CZ								3.840		2.972	2			
H12	NE2	R39	NE										3.034	1			
H12	NE2	R39	NH1										3.567	1			
H12	NE2	R39	NH2								3.228		3.172	2			
H12	NE2	K41	NZ		2.795									1			
H12	NE2	K41	CD		3.347									1			
H12	NE2	K41	CE		3.355									1			
H12	NE2	T45	O			3.804								1			
H12	NE2	T45	OG1								2.947			1			
H12	NE2	F46	CE1									3.937		1			
H12	NE2	V118	C				3.613							1			
H12	NE2	H119	N				3.967							1			
H12	NE2	H119	CA				3.648							1			

S-peptide		S-protein		distance after simulation [Å]										atom pair	S-peptide residue		
residue	atom	residue	atom	model 01	model 02	model 03	model 04	model 05	model 06	model 07	model 08	model 09	model 10	contact counts	contact positions	sum of contacts	position-to-sum ratio
H12	NE2	F120	N										3.826	1			
H12	NE2	F120	CB		3.723			3.821						2			
H12	NE2	F120	CG		3.586			3.865						2			
H12	NE2	F120	CD1		3.418									1			
H12	NE2	F120	CD2					3.909						1			
H12	NE2	F120	CE1								3.777			1			
M13	CB	S21	N									3.755		1	25	29	1,16
M13	CE	V47	C					3.809						1			
M13	CE	E49	O		3.199						3.181	3.695		3			
M13	CE	S50	C			3.549								1			
M13	CE	S50	O			3.784								1			
M13	CE	L51	N			3.383								1			
M13	CE	L51	CA			3.538								1			
M13	CE	L51	CD1				3.659							1			
M13	CE	V54	CB					3.861						1			
M13	CE	V54	CG1			3.627		3.516	3.753					3			
M13	CE	V54	CG2										3.957	1			
M13	CG	R33	CZ			3.786								1			
M13	CG	R33	NH1			3.474								1			
M13	CG	R33	NH2			3.514								1			
M13	CG	V47	CB								3.981			1			
M13	CG	V47	CG2	3.797										1			
M13	CG	H48	NE2					3.913						1			
M13	CG	H48	CD2					3.969						1			
M13	CG	V54	CB	3.706										1			
M13	CG	V54	CG1	3.385										1			
M13	SD	V47	CB									3.785		1			
M13	SD	S50	OG	3.664										1			
M13	SD	S50	CA	3.945										1			
M13	SD	S50	CB	3.950										1			
M13	SD	V54	CG1			3.871								1			
D14	CB	S21	N									3.876		1	51	63	1,24
D14	CB	M29	SD			3.811								1			
D14	CB	R33	NE		3.976			3.702				3.998		3			
D14	CB	F46	CZ	3.858										1			
D14	CB	V47	O								3.560			1			
D14	CG	S21	CA									3.957		1			
D14	CG	S21	N									3.525		1			
D14	CG	M29	CE			3.893								1			
D14	CG	M29	SD			3.922								1			
D14	CG	R33	NE					3.587						1			
D14	CG	F46	CG							3.978				1			

S-peptide		S-protein		distance after simulation [Å]										atom pair	S-peptide residue		
residue	atom	residue	atom	model 01	model 02	model 03	model 04	model 05	model 06	model 07	model 08	model 09	model 10	contact counts	contact positions	sum of contacts	position-to-sum ratio
D14	CG	F46	CZ	3.839										1			
D14	CG	T82	CB			3.903								1			
D14	CG	T82	OG1										3.380	1			
D14	OD1	M29	CE			3.923								1			
D14	OD1	R33	NE	3.168				2.967						1			
D14	OD1	R33	CZ	3.218										1			
D14	OD1	R33	NH2	3.063										1			
D14	OD1	R33	CD	3.947				3.448						2			
D14	OD1	R33	CG					3.500						1			
D14	OD1	F46	CB			3.134							3.323	2			
D14	OD1	F46	CG			3.589								1			
D14	OD1	F46	CD2			3.285								1			
D14	OD1	T82	CB										3.679	1			
D14	OD1	T82	OG1										3.379	1			
D14	OD1	T82	CG2										3.806	1			
D14	OD2	S21	CA									3.619		1			
D14	OD2	S21	C									3.276		1			
D14	OD2	S21	N									3.677		1			
D14	OD2	T25	OH					3.442			3.878			2			
D14	OD2	T25	CE2						3.778					1			
D14	OD2	T25	CD2									3.841		1			
D14	OD2	T25	CB							3.916				1			
D14	OD2	M29	CG					3.701			3.639			2			
D14	OD2	R33	NH2	3.510										1			
D14	OD2	R33	CZ	3.604										1			
D14	OD2	R33	NH1	3.723										1			
D14	OD2	H42	CG			3.371								1			
D14	OD2	F46	CZ	3.232										1			
D14	OD2	V47	C				3.754							1			
D14	OD2	H48	N				3.688							1			
D14	OD2	H48	CA				3.442							1			
D14	OD2	H48	CB			3.654	3.532							2			
D14	OD2	H48	CG				3.507							1			
D14	OD2	H48	CD2			2.924	3.268		3.033					3			
D14	OD2	H48	CE1					3.248						1			
D14	OD2	H48	NE2					3.895	3.486					2			
D14	OD2	F46	CE1	3.096										1			
D14	OD2	T82	CB			3.776	3.473							2			
D14	OD2	T82	OG1			2.882	2.683							2			
D14	OD2	T82	CG1							3.044				1			
S15	CA	R33	NH1					3.923						1	15	20	1,33
S15	CB	M29	SD			3.953								1			

S-peptide		S-protein		distance after simulation [Å]										atom pair	S-peptide residue		
residue	atom	residue	atom	model 01	model 02	model 03	model 04	model 05	model 06	model 07	model 08	model 09	model 10	contact counts	contact positions	sum of contacts	position-to-sum ratio
S15	CB	R33	NH1					3.533						1			
S15	CB	R33	NH2				3.822		3.615					2			
S15	CB	E49	C							3.793				1			
S15	CB	S50	CA							3.624				1			
S15	CB	S50	N							3.829				1			
S15	OG	M29	C								3.792			1			
S15	OG	M29	CA								3.782			1			
S15	OG	M29	CG			3.694								1			
S15	OG	R33	CZ						3.636					1			
S15	OG	R33	OG				3.413	3.744						2			
S15	OG	R33	NH1				3.396		3.793			3.647		3			
S15	OG	R33	NH2				2.648		2.722					2			
S15	OG	E49	C							3.637				1			

a) contacts between paired atoms are assumed when distances are < 4 Å

b) atoms with distances > 4 Å to next neighbor atoms are not listed.

c) atoms from amino S-peptide acid residues 16-20 are not considered
